# Choindroitinase ABC I-Mediated Enhancement of Oncolytic Virus Spread and Anti Tumor Efficacy: A Mathematical Model

**DOI:** 10.1371/journal.pone.0102499

**Published:** 2014-07-21

**Authors:** Yangjin Kim, Hyun Geun Lee, Nina Dmitrieva, Junseok Kim, Balveen Kaur, Avner Friedman

**Affiliations:** 1 Department of Mathematics, Ohio State University, Columbus, Ohio, United States of America; 2 Department of Neurological Surgery, Ohio State University, Columbus, Ohio, United States of America; 3 Mathematical Biosciences Institute, Ohio State University, Columbus, Ohio, United States of America; 4 Department of Mathematics, Korea University, Seoul, Republic of Korea; 5 Department of Mathematics, Konkuk University, Seoul, Republic of Korea; University Hospital of Navarra, Spain

## Abstract

Oncolytic viruses are genetically engineered viruses that are designed to kill cancer cells while doing minimal damage to normal healthy tissue. After being injected into a tumor, they infect cancer cells, multiply inside them, and when a cancer cell is killed they move on to spread and infect other cancer cells. Chondroitinase ABC (Chase-ABC) is a bacterial enzyme that can remove a major glioma ECM component, chondroitin sulfate glycosoamino glycans from proteoglycans without any deleterious effects in vivo. It has been shown that Chase-ABC treatment is able to promote the spread of the viruses, increasing the efficacy of the viral treatment. In this paper we develop a mathematical model to investigate the effect of the Chase-ABC on the treatment of glioma by oncolytic viruses (OV). We show that the model's predictions agree with experimental results for a spherical glioma. We then use the model to test various treatment options in the heterogeneous microenvironment of the brain. The model predicts that separate injections of OV, one into the center of the tumor and another outside the tumor will result in better outcome than if the total injection is outside the tumor. In particular, the injection of the ECM-degrading enzyme (Chase-ABC) on the periphery of the main tumor core need to be administered in an optimal strategy in order to infect and eradicate the infiltrating glioma cells outside the tumor core in addition to proliferative cells in the bulk of tumor core. The model also predicts that the size of tumor satellites and distance between the primary tumor and multifocal/satellite lesions may be an important factor for the efficacy of the viral therapy with Chase treatment.

## Introduction

Glioblastoma is one of the most aggressive type of brain cancer with the median survival time of approximately one year [1], [2]. It is characterized by rapid proliferation and high invasion. Glioma are resistant to radiotherapy and chemotherapy and eventually recurs [3]. Oncolytic viruses (OV) are genetically manipulated viruses that can destroy cancer cells but do minimal damage to normal healthy tissues [4]. These viruses can preferentially replicate in tumor cells, leading to their lytic destruction. Accompanying cell lysis the virus particles burst out and proceed to spread and infect other cancer cells. While the world's first OV, H101 (Oncorine, an OV functionally identical to ONYX-015), was approved by the Chinese State Food and Drug Administration, the regulatory approval of OVs in the United States and Europe is pending the results of randomized and large phase III studies [5]. Despite the great potential and regulatory approval of OV for clinical use in China, its inefficient dispersal property within the tumor ECM has been recognized as a major barrier for its anti-tumor efficacy [5]. Tumor ECM plays a pivotal role in inhibiting virus spread [6]–[11] and leads to limited viral replication and reduced cytolytic efficacy [12]. Major ECM components blocking this viral dispersal in the neural ECM include hyaluronic acid (HA) [13], [14] and proteoglycans [11], [14] and these structural components are also known for hindering large therapeutic molecules [11].

The use of ECM degradating enzymes could significantly increase the efficacy of OV treatment by enabling virus to move more freely among uninfected tumor cells. Unlike ECM in normal tissue, tumor ECM continuously undergoes remodeling and extensive synthesis [15]–[17]. ECM remodeling is also an important part of brain tumor angiogenesis [18], [19]. The brain ECM contains mainly macromolecules such as glycosaminoglycans (GAGs) and proteoglycans (PGs) with a smaller fraction of fibrillary glycoproteins such as collagens, fibronectin, or elastin [14], [19], [20]. Tumor ECM inhibits penetration of anti-tumor agents, leading to low therapeutic efficacy. High interstitial fluid pressure inhibits delivery of the agents by convection through the tumor interstitial matrix resulting in a rather passive diffusion as the major mode of transport of macromolecules [21], [22]. Tumor penetration of macromolecules such as IgG is negatively correlated with increased collagen content [21], [23]. Use of collagenase, an enzyme that breaks the peptide bonds in collagen, was shown to increase the diffusivity of macromolecules (IgG) [23], to reduce interstitial fluid pressure, and to enhance the transvascular transport through convection [24].

Protease- or hyaluronidase-mediated digestion of the ECM can enhance intratumoral penetration [25], and MMP-enhancing oncolytic relaxin is known to increase anti-tumor efficacy of virus spread [7]. Relaxin is a peptide hormone that is able to reduce the synthesis and secretion of interstitial collagens and increase the level of metalloproteinase (MMP) and oncolytic adenovirus expressing relaxin was shown to promote OV dispersal through the tumor ECM [7]. However, ECM degrading enzymes need to be used with caution since intracranical use of degrading enzymes can cause serious complications. For example, hemorrhagic necrosis of brain can be induced by collagenase-mediated ECM disruption, brain proteases are associated with neurodegenerative diseases [26], and hyaluronidase can promote optic glioma growth through astrocytic reactivity. [27].

Secreted and membrane-bound chondroitin sulfate proteoglycans (CSPG) linked to extracellular hyaluronan is one of major components of the ECM in the brain [13]. CSPGs include versican, brevican, phosphacan, and NG2. CSPGs also play a role as axon growth inhibitory molecules that are present in the glial scar, and are responsible in part for regeneration failure of scar after damage to the CNS [28]. In CNS tumors, increased expression of CSPGs is associated with tumor growth, invasion, and angiogenesis [29], and is responsible for diffusion-limiting properties [11]. Accumulated CSPGs in the glioma ECM increase tortuosity of the extracellular space and interstitial pressure within the tumor [11], leading to poor transport of large therapeutic agents [22]. Hence enzymatic manipulation, lowering the CSPG levels, would be a way of improving interstitial transport of therapeutic agents into the tumor. In this connection, ECM macromolecules have been considered as potential therapeutic targets for adjuvant therapy [30], [31]. For example, tumor proliferation and dispersion were inhibited when these molecules were reduced or inhibited by blocking antibodies against versican [30] or interfering RNA against phosphacan [32]. CSPGs can also be degraded by MMP-1 and MMP-8, leading an increase in hydraulic conductivity and particle diffusion in solid tumors [33].

**Figure 1:**
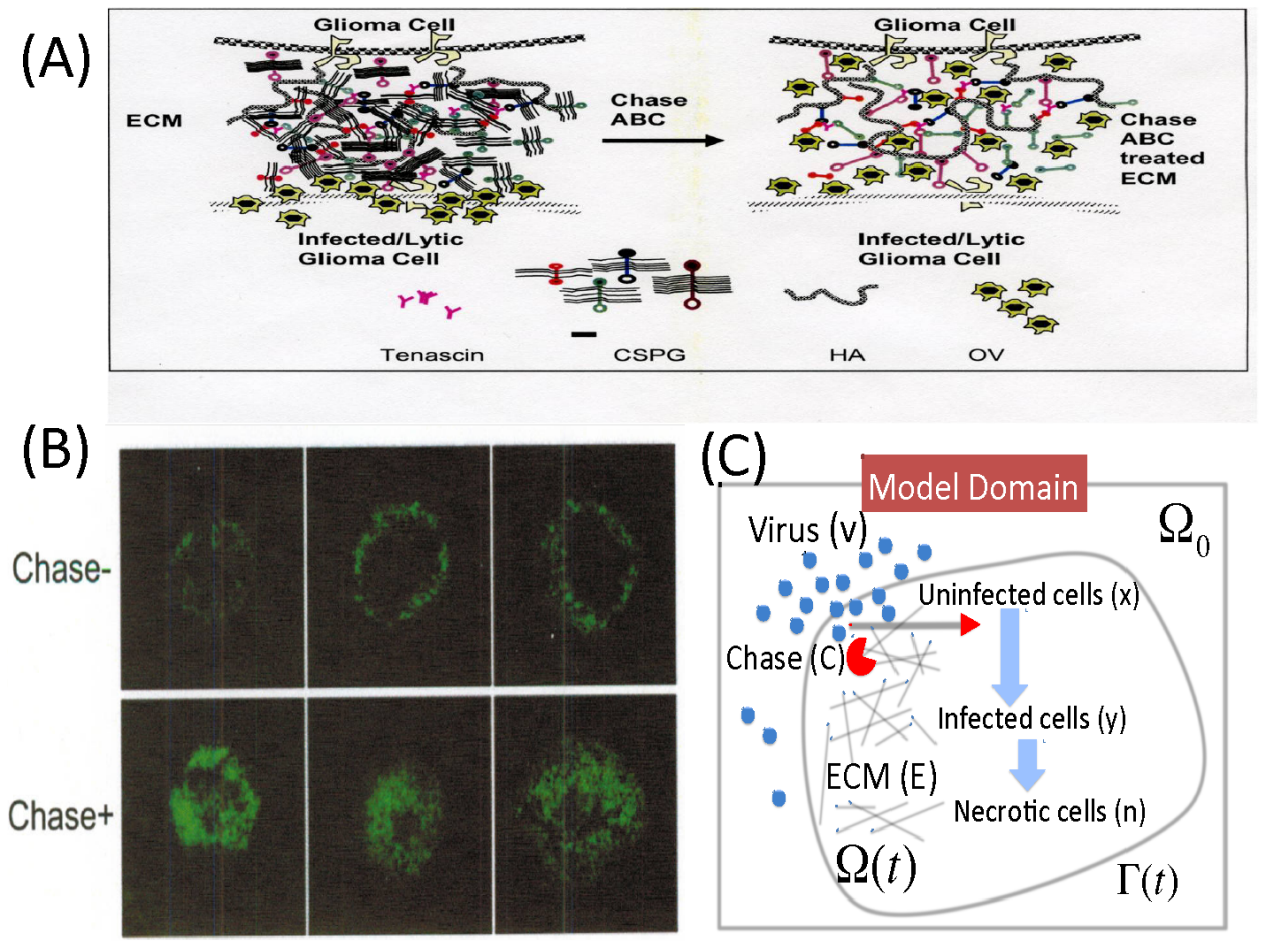
Overview of ECM degradation in experiments and schematic of the mathematical model. (A) A schematic of ECM degradation and Chase ABC treated ECM. (B) In experiments [11], viral spread was evaluated 4 days after OV treatment through detection of the GFP-positive infected cells. Images in upper and lower panels show 3 representative spheroids (n = 6/group) treated with vehicle (Chase-) and Chase-ABC (Chase+), respectively (Figure 1D in [11]). Chase-treated viruses spread more efficiently throughout the spheroid compared to control-treated spheroids. Glioma U87
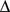
EGFR spheroids were cultured on organotypic brain slices for 72 hours followed by treatment with purified Chase-ABC or vehicle for 24 hours before or after infection with rQNestin34.5. (with permission from the following article: *Dmitrieva N, Yu L, Viapiano M, Cripe TP, Chiocca EA, Glorioso JC, Kaur B., Chondroitinase ABC I-mediated enhancement of oncolytic virus spread and antitumor efficacy, Clin Cancer Res, 17(6):1362-72 (2011)*. (C) A schematic of the mathematical model. Three types of glioma cells (uninfected (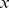), infected (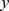), and dead (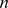) cells) and ECM component (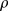) consist of a growing tumor mass with a free boundary 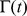. Viruses (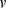) from outside (or inside) the tumor mass penetrates through the network of ECM components within the the tumor mass to infect glioma cells (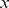). Diffused Chase-ABC (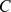) degrades ECM components and help virus infiltrate the narrow intercellular space between cells within the tumor mass.

Chondroitinase ABC I (Chase-ABC) is a bacterial enzyme that can remove Chondroitin sulfate glycosoamino glycans from proteoglycans without any deleterious effects *in vivo* [11] and has been studied for its effect on neuronal regeneration after injury. Chase-ABC has been widely used to enhance regeneration of injured axonal tracts due to its a long-lasting 'loosening' effect on the ECM scaffold [28], [34]. Based on extensive positive evidence from preclinical models, Chase-ABC I have recently been used in Phase I/II trials for treatment of patients in Japan [35]. In an effort to investigate the use of bacterial chondroitinase to enhance anticancer therapy, Dmitrieva *et al*. [11] examined the effect of Chase-ABC on tumor ECM, OV spread, and efficacy. In the experiments, three-dimensional glioma spheroids placed on cultured brain slices were utilized to evaluate OV spread; see Figure 1A. Replication-conditional OV expressing Chase ABC (OV-Chase) was tested for spread and efficacy in glioma spheroids and glioma xenografts were utilized to compare anti-tumor efficacy of OV-Chase, HSVQ (control) and PBS. Dmitrieva *et al*. [11] found that OV spread in glioma spheroids grown on brain slices were significantly enhanced by Chase-ABC treatment, that cell migration or invasion were not enhanced by OV Chase treatment. Together these results suggest that degradation of ECM by OV expressed bacterial Chase-ABC was the key factor in enhancing OV spread and anti tumor efficacy. It has been shown that virus spreads more efficiently in spheroids compared to control-treated spheroids when they are treated with Chase [11]; see Figure 1B.

Kim *et al*. [36] recently developed a mathematical model of invasive glioma cells in 3D tumor spheroids. The model was shown to be capable of reproducing migration patterns of glioma cells in *in vitro* experiments, exhibiting, in particular, dispersion and branching of cells. The model included MMP activity and glucose levels as well as chemotaxis, haptotaxis and cell-cell adhesion forces. The rapid migration of cells is caused primarily by the chemotaxis forces including glucose levels. There are several mathematical models of brain tumors based on reaction-diffusion process [37]–[41], cellular automata [42], the transition between migration and proliferation using kinetic or diffusion models [43]–[45], and intracellular signaling pathways [1], [46], [47]. A general review on mathematical models of tumor growth can be found in [48], [49].

In this paper we develop a mathematical model based on the schematic diagram of Figure 1C. The model consists of a coupled system of partial differential equations involving the following variables:
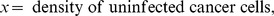


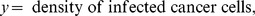


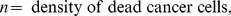


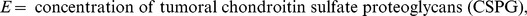


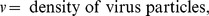


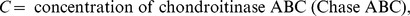


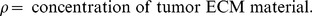



It will be shown that the model simulations for a spherical glioma are in qualitative agreement with experimental results of [11]. It is a common practice to inject the virus into serval different locations within the tumor. The mathematical model can be used to determine preferred locations for such injections. In Materials and Methods section, we present the mathematical model, and in Results Section we simulate the model, and then also perform several experiments and propose some hypotheses. Parameter estimation, nondimensionalization of the model and numerical scheme of dimensionless governing equations are given in Text S1.

## Materials and Methods

In this section we develop a system of partial differential equations with initial and boundary conditions, for the variables introduced in Section 1, in a spherical tumor 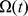 and boundary 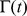.

### Cell density (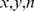)

In order to describe the time evolution of densities of different types of tumor cells (uninfected, infected, and dead cells), we take into account cell proliferation of tumor cells and the virus infection of tumor cells, followed by the clearance process, and the passive movement due to the velocity field 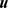 generated from tumor growth; in Section 2.6, we will derive an equation for 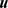. The equations for 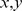 and 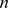 are taken from the model by Friedman *et al*. [50];
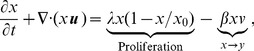
(1)

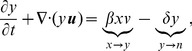
(2)

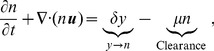
(3)where 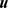 is the velocity, 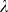 is the proliferation rate of uninfected tumor cells, 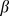 is the infection rate, 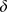 is the infected cell lysis rate, and 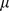 is the removal rate of dead cells. We note that treatment by Chase-ABC does not affect these equations. We also note that Friedman *et al*. [50] included the effect of the immune system and immune suppressive treatment on the efficacy of the OV injection; in the present paper we ignore this aspect of the treatment, concentrating only on the efficacy derived from Chase-ABC treatment.

### Concentration of tumoral chondroitin sulfate proteoglycans (CSPG) (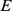)

Secreted and membrane-bound chondroitin sulfate proteoglycans (CSPG), described below in Section 0, linked to extracellular hyaluronan form a major component of the ECM in the brain [13]. It was found that CSPGs detected in glioma cell lines and tumor samples interfere with OV spread in glioma spheroids [11]. The ability of Chondroitinase ABC (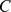) to degrade the tumoral chondroitin sulfate proteoglycans (CSPG) is the key regulation factor in this paper. As in [11], we hypothesize that Chase-ABC-mediated digestion of glioma CS-GAGs degrades glioma ECM and opens up space for viruses to diffuse faster.

The conservation of mass for the CSPG concentration gives
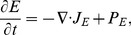
(4)where 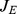 is the flux for mobility and 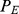 is the CSPG production/death rate. The CSPG is convected by the fluid velocity 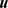,
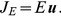
(5)


CSPG degradation rate is given by
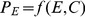
(6)where 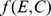 describes the degradation of the ECM (CSPG) by Chondroitinase ABC (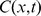).

Chase-ABC degrades the key ECM component CSPG(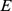). We assume that the ECM degradation rate is proportional to both the ECM concentration and Chase concentration,
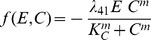
(7)where 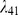 is a degradation rate of the ECM component, CSPG.

In summary, we have the following governing equation for the CSPG concentration
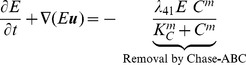
(8)


This equation describes the time evolution of the CSPG density after Chase-ABC-assisted degradation of CSPG with the passive movement from the velocity 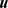.

### Density of free virus particles (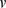)

In this section we describe the movement and replication/decay process of virus particles within the complex ECM structure, CSPG, in the microenvironment. Treatment with Chase-ABC improves viral spread [11]. We assume the virus can spread in CSPG concentration-dependent manner and duplicate and undergoes apoptosis. From conservation of mass for density of free virus particles (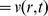), we get
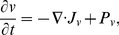
(9)where 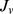 is the flux for mobility and 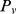 is the birth/death rate. The only contribution to the flux 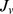 is flux from random motility of the free viruses:
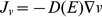
(10)where 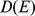 is the random motility that depends on ECM (CSPG) concentration 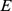 and quantifies how virus particles outside infected cells can freely move around in less dense ECM (CSPG). We use the following phenomenological form
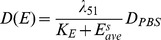
(11)where 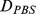 is the diffusion coefficient in the PBS (reference) case, 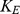 is an inhibition parameter, 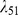 is a scaling factor, and 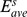 is the average local density of the ECM concentration over a local neighborhood, *i.e.*,
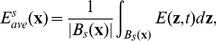
(12)where 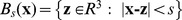 is the ball in 3D (or a disc in 2D) with center 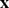 and a *sensing radius* 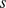, and 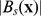 is the volume of 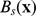. Here we use the convention that 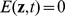 if 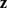 is outside the tumor 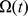. When 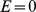, this gives 
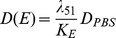
.

We assume free virus particle can replicate with replication rate 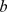 in the presence of infected cells (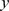) with lysis (or death) rate 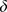 within the growing tumor [50]

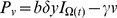
(13)where 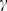 is the clearance rate of viruses, and 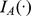 is the indicator (characteristic) function over a domain 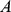.

In summary, we have the following governing equation for the virus density,
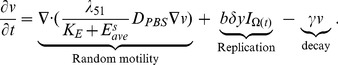
(14)


### Chondroitinase ABC (Chase-ABC; 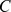)

We next derive the time evolution of Chase-ABC concentration in response to injection, secretion and ECM degradation, and natural decay, in the system. From conservation of mass for the concentration of Chase-ABC (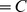), we get
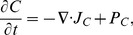
(15)where 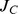 is the flux and 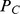 is the production/decay rate of the Chase-ABC. The only contribution to the flux 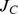 is flux from the diffusion process:
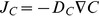
(16)where 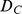 is the diffusion coefficient of the Chase-ABC. The Chase-ABC is applied in the far away field and degrades the key ECM component CSPG (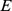). Chase-ABC may be secreted by infected tumor cells through virus [51] or be directly applied to the field surrounding a growing tumor in addition to the injection of virus [11]. Taking into account the natural decay of the molecule, we set
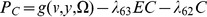
(17)where 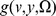 is a function that describes a production of Chondroitinase, which depends on virus, infected tumor cells, and possibly other factors in the tumor 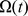, 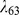 is the ECM degradation parameter, and 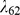 is the decay rate.

In summary, the governing equation for the Chase-ABC is as follows:
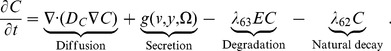
(18)


### Tumor ECM material (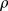)

Glioma cells destabilize the normal cell-matrix structure by overexpressing or generating their own altered neural ECM molecules [52], [53], creating a disorganized ECM structure that promotes tumor-cell movement and proliferation [13]. We assume that only proliferating uninfected tumor cells (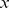) secrete tumor ECM materials that undergo remodeling. By conservation of mass, we get
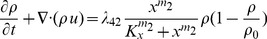
(19)where 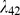 is secretion rate of ECM material by uninfected tumor cells, 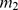 is the Hill-type function exponent, 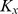 is the Hill-type function parameter, 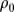 is the maximum capacity of the tumor ECM material.

### Growing spheroid

The tumor spheroid either grows or shrinks in response to growing mass from proliferative cells and added ECM materials, and decreased mass from the ECM degradation process. We model this process by the Stokes equation with added material acting as added pressure:
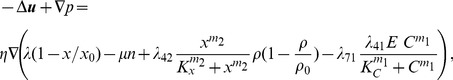
(20)where 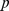 is the pressure and 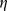 is the viscosity of fluid. The first two terms, third, and fourth terms on the right-hand side of equation (20) represent additional total change in the density of cells, new material of the ECM, and degraded ECM (CSPG) components, respectively. By conservation of mass, 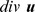 is equal to the expression in parenthesis on the right-hand side of equation (20).

#### Boundary Condition

The governing equations (1)–(20) is coupled with the following boundary conditions
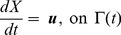
(21)

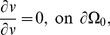
(22)

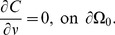
(23)where 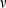 is the outer normal vector and 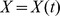 represents the free boundary. Parameter and reference values are given in Tables 1 and 2.

**Table 1:** Model parameters.

Parameter	Description	Dimensional value	Ref
Diffusion coefficients/Random motility			
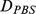	Random motility of virus (PBS)	3.89 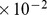 ( 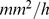 )	[89], tw
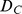	Diffusion coefficient of Chase	3.89 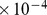 ( 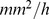 )	tw
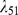	virus random motility controlling parameter	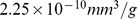	tw
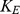	Inhibition parameter of virus motility from ECM	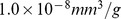	tw
Production/remodeling rates			
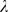	Proliferation rate of tumor cells	1.54 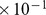 ( 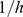 )	[50], tw
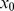	carrying capacity of uninfected tumor cells	= *x* *	tw
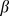	Infection rate	7.0 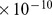 ( 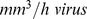 )	[50]
b	Burst size of infected cells	50 ( 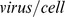 )	[50]
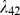	ECM production rate from uninfected tumor cells	6.0 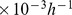	[36], tw
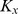	Hill-type coefficient in tumor ECM remodeling/reconstruction by uninfected tumor cells	= *x* *	tw
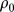	carrying capacity of tumor ECM	= *ρ* *	[36]
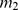	Hill-type parameter in ECM remodeling/reconstruction by uninfected tumor cells	1†	tw
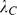	Secretion rate of ECM degrading enzyme	3.0 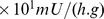	tw
Inhibition/degradation/decay rates			
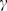	Clearance rate of viruses	1.8 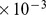 ( 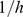 )	[50]
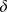	Infected cell lysis rate	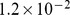 ( 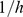 )	[50], tw
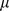	Removal rate of dead cells	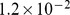 ( 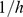 )	[50], tw
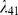	ECM degradation rate	9.0 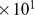 ( 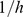 )	tw
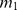	Hill-type parameter in ECM degradation by Chase	1†	tw
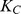	Hill-type coefficient in ECM degradation by Chase	= *C* *	tw
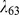	Chase reaction rate due ECM degradation	6.0 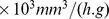	tw
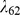	Natural decay rate of Chase	6.0 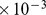 ( 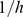 )	[28], tw

Units are indicated in parenthesis () in the third column.

† = dimensionless value.

*tw = estimated in this work.

**Table 2:** Reference value used in the model.

Var	Description	Dimensional value	Ref
L	Length scale	3 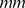	[11]
T	Time scale	1.67 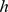	tw
D	Characteristic diffusion coefficient	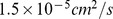	tw
x*	Uninfected cell density	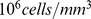	[50], [92]
y*	Infected cell density	= x*	[50], [92]
n*	Dead cell density	= n*	[50], [92]
v*	virus concentration	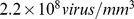	[50], [92]
ρ*	Concentration of other ECM materials	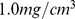	[100], [101]
E*	ECM (CSPG) concentration	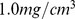	[94]–[100]
C*	Concentration of Chase-ABC	C* = 50*mU*/*ml*	[28], [34], [93]

tw = estimated in this work.

## Results

In next two sections we take 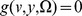.

### Results and comparison with experimental data

**Figure 2:**
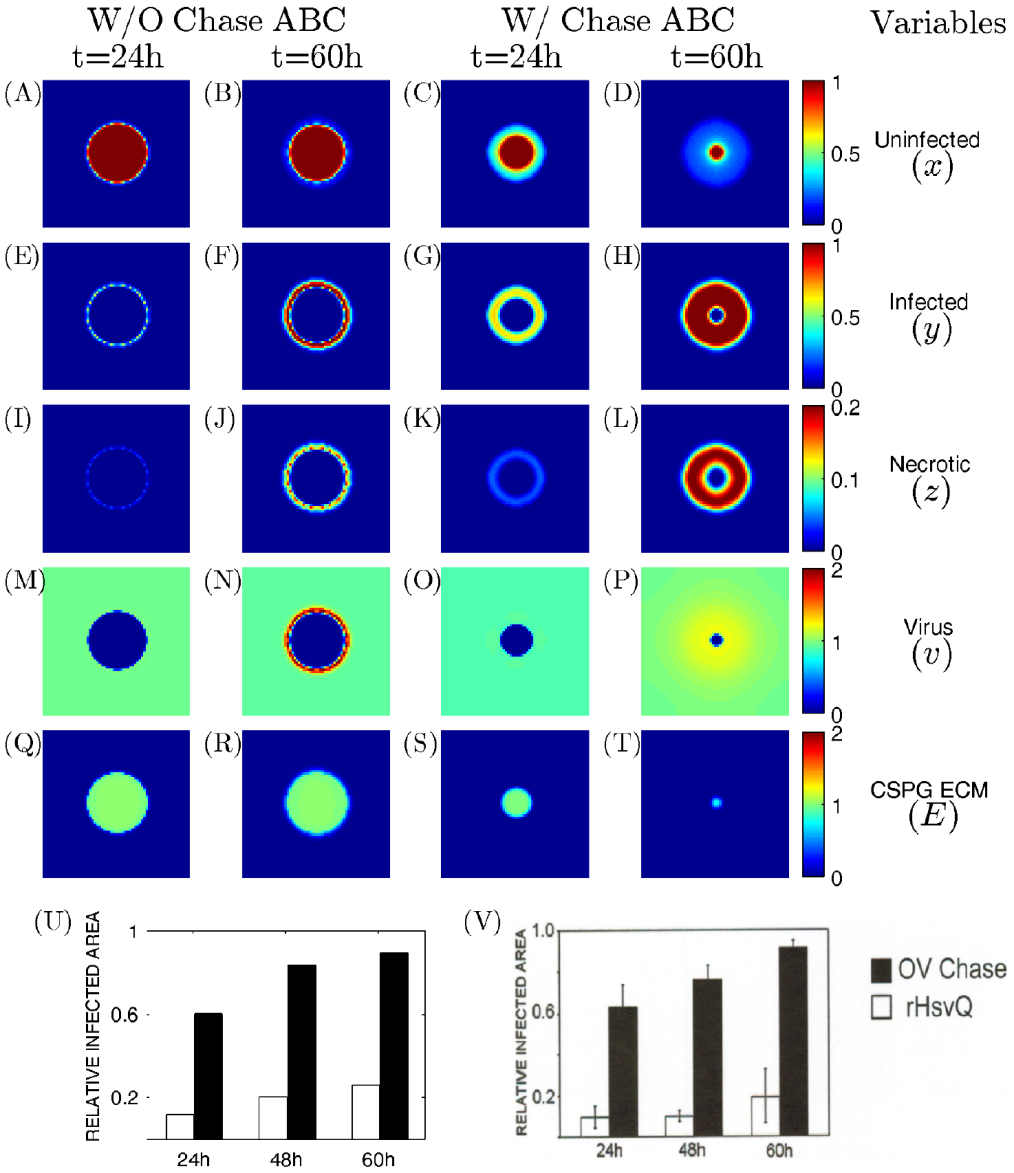
Dynamics of the model and comparison with experimental data. (A-T) Dynamics of the model in the control case (without chase treatment) and with chase treatment. Profiles of densities of uninfected, infected, and dead cells, virus, and CSPG ECM, are shown at 24, and 60 hours postinfection. Without chase treatment, virus spread is limited due to thick ECM components. With Chase treatment, ECM degradation is improved, leading to enhanced virus spread. The enclosing box size is 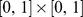. 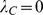. (U, V) OV-Chase has improved virus spread in glioma spheroids. Relative infected areas (*i.e.*, up to scale) at 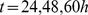 were calculated from model simulations (U) and experiments (V). In simulations, viral spread was calculated as the infected area relative to the total area of the glioma spheroid. The black and white bars represent the results with and without ChaseABC, respectively. 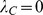. In experiments, viral spread was calculated as the GFP-positive area relative to the total area of the spheroids in [11].

We first compare our simulation results with experimental data in [11]. In experiments [11], various human glioma cell lines (U343, U87, U87DEGFR, LN229, Gli36DEGFR-H2B-RFP, X12) and Vero cells were used in *in vitro* and *in vivo* expriments. OV-Chase is an oncolytic HSV-1-expressing bacterial Chase-ABC driven by a promoter within the backbone of rHsvQ (control). In vitro studies, in order to see the spread of rHsvQ and OV-Chase in 3-dimensional glioma spheroids on brain slices, the spheroids were infected with 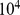 pfu (plaque forming units) of rHsvQ or OV-Chase. Then, infected areas for the cases of Chase(−) and Chase(+) were calculated by both visualization and quantification of infected cells by appearance of OV-encoded GFP over time. In *in vivo* studies, the glioma cells implanted stereotactically at a position 2 mm lateral to bregma at a depth of 3 mm for animal studies and animals were inoculated with control (PBS) or virus in the absence or presence of Chase-ABC ten days after tumor cell implantation. Intratumoral injections of HBSS or the indicated OV was performed for the mice bearing subcutaneous tumors with the initial average volume of 80 to 150 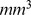. Seventy-two hours after viral infection, the amount of infectious viral particles and infected areas were quantified by a standard method. Figures 2A-2L show spatial profiles of uninfected cells (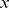), infected cells (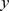), dead cells (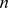), virus (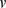) and CSPG ECM (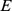) at 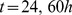 without and with Chase treatment. Without Chase treatment, CSPG ECM degradation is inhibited (Figures 2Q,2R) and virus spread (Figures 2M,2N) is limited due to thick ECM components. Figures 2M,2N show localized virus in the annulus subdomain. Infected tumor cells and dead cells are localized at the periphery of the spheroid due to this inhibited virus spread toward the center of the spheroid. There are large portion of uninfected tumor cells at the center of the tumor spheroid. On the other hand, Chase treatment promoted CSPG ECM degradation and decomposition (Figures 2S,2T) and led to fast and effective spread of virus (Figures 2O,2P) and more infection of tumor cells near the spheroid center (Figures 2G,2H). One also observes significant reduction in uninfected tumor cell density (Figures 2C,2D) and widespread distribution of dead tumor cells at the center of the tumor spheroid (Figures 2K,2L).

In order to compare the computational results with experimental data, we calculated the relative infected area using density distribution of infected tumor cells *i.e.*, we use the infected area relative to the total area of the glioma spheroid. Figure 2U shows relative infected areas with (black) and without OV-Chase treatment. This result is in good qualitative agreement with experimental data in [11] shown in Figure 2V. The experiments in [11] and the simulations in this paper demonstrate that OV-chase treatment improve the infection of virus within the tumor thus decreasing the burden of cancer mass.

### The role of the microenvironment

**Figure 3:**
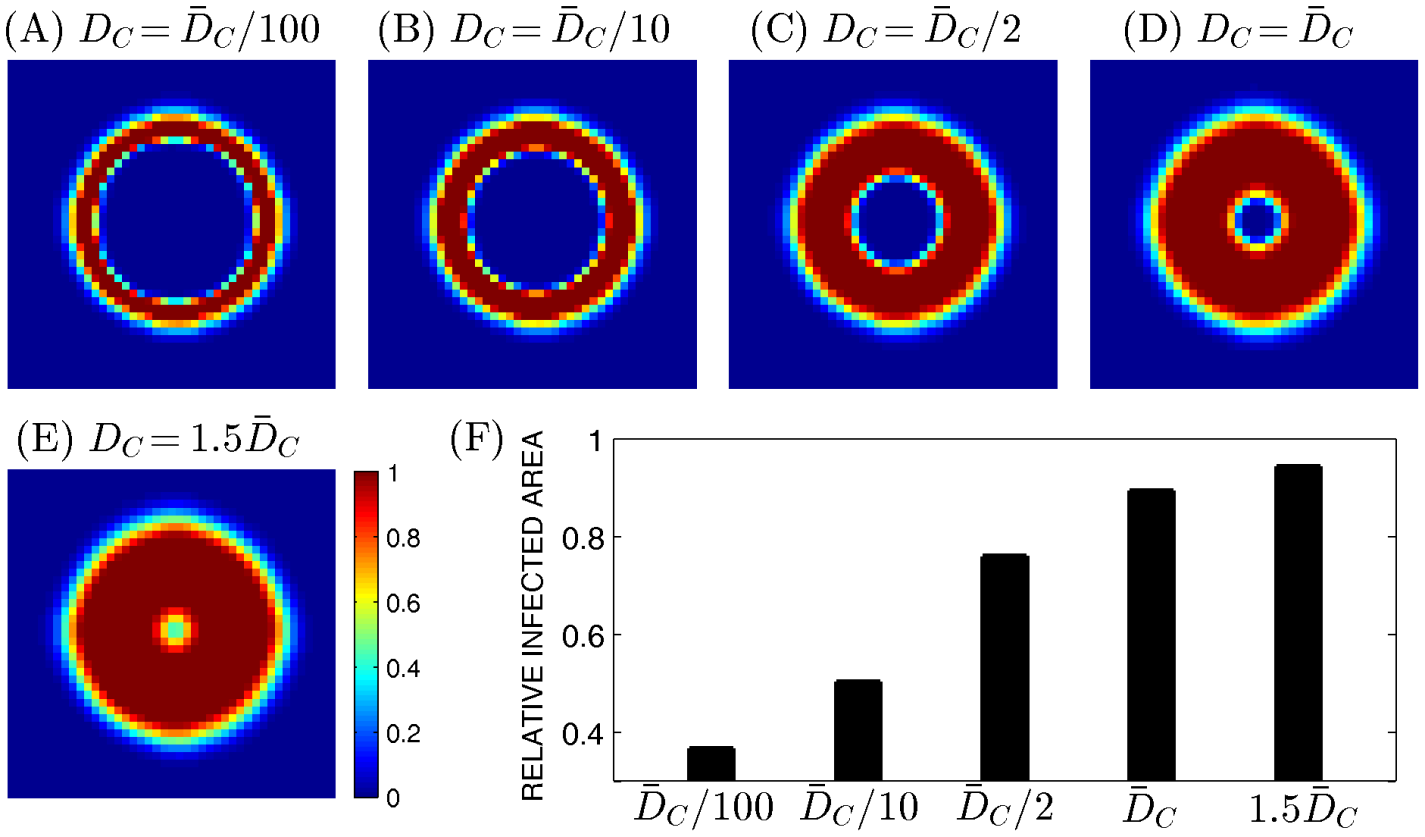
Effect of Chase diffusion on virus infection. (A-E) Viral spread were shown at 60 hours postinfection for various values of 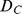 (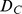 = 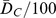 in (A), 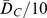 in (B), 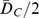 in (C), 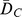 in (D), 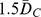 in (E)). The default value of 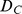, 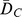, is 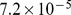. The enclosing box size is 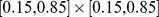. (F) Relative infected area for each case in (A-E).

The microenvironment plays a significant role in regulating the spread and treatment efficacy of many types of cancer [36], [54], [55] including glioblastoma [3]. Chase diffusivity which clearly affects the tumor microenvironment may depend on many factors such as composition of ECM materials in *in vivo* system. In Figure 3, we investigated effect of diffusivity of Chase on virus spread. The relative infected area is increased when diffusivity of Chase is increased. Almost all tumor cells at the center of tumor mass were infected when diffusivity is 1.5-fold larger (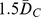) than was in the control case (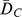). On the other hand, when diffusivity of Chase was decreased, the virus spread was severely impaired. For example, the infected area was decreased by 50% and 37% and only the cells on the periphery of the tumor mass were infected when the diffusion coefficient of Chase (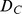) is 10- and 100-fold smaller, respectively. This implies that virus spread would be significantly less effective when a tumor is located in a harsh microenvironment where ECM component may be densely packed or quickly remodeled.

**Figure 4:**
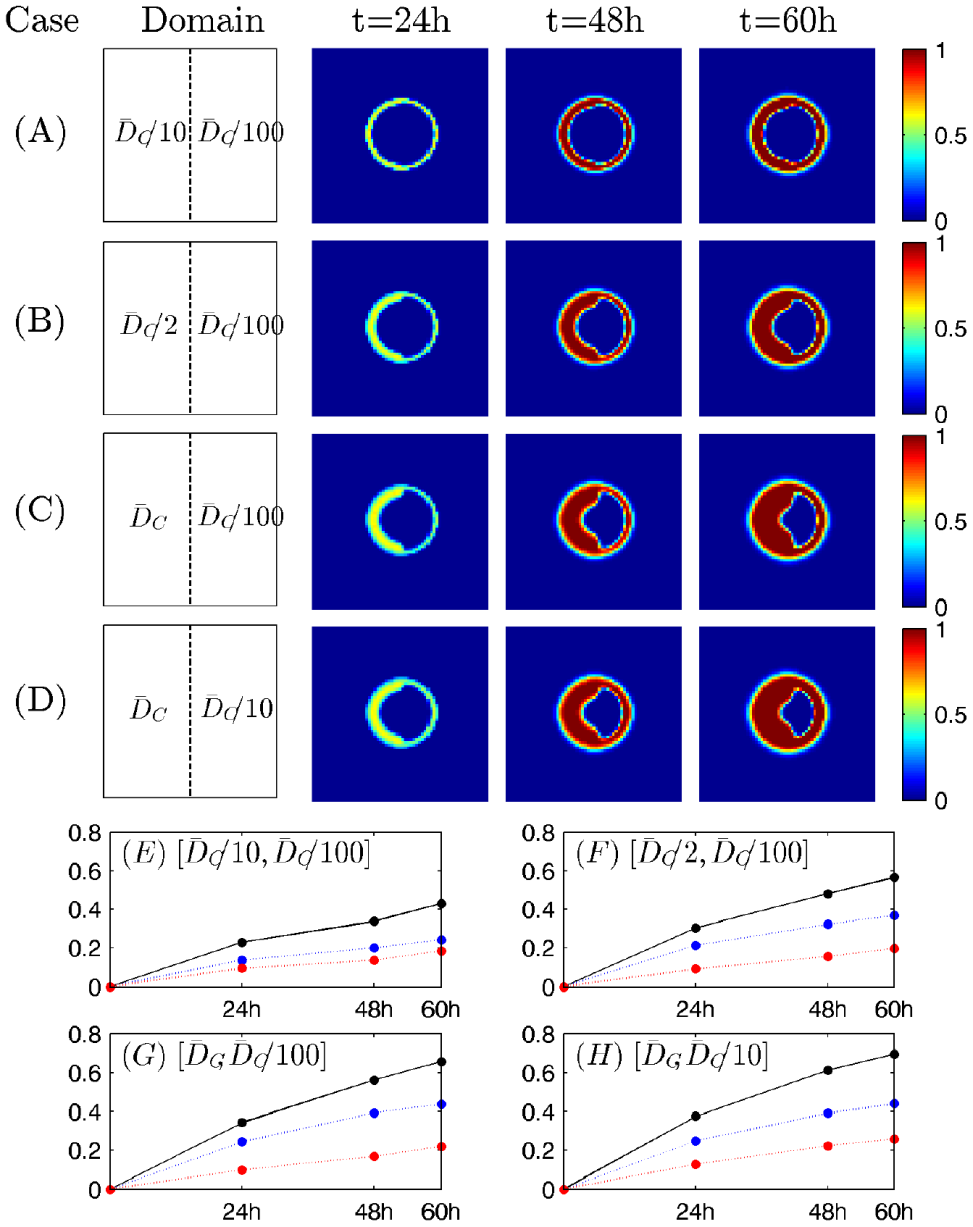
The role of the microenvironment in regulating the virus spread. (A-D) Virus spreads were shown at 24, 48, and 60 hours postinfection when a tumor was imposed in the different microenvironment. Slower transport of virus in a harsh microenvironment (slower diffusion) decreases virus spread efficacy. Different diffusion coefficients of 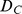 were prescribed on the left (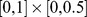) and right (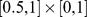) half of the domain 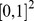 ((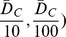 on the left and right in (A); (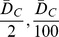) on the left and right in (B); (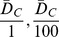) on the left and right in (C); (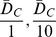) on the left and right in (D);). The enclosing box size is 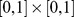. The relative infected areas at 60 hours of (A), (B), (C), and (D) are 0.4397, 0.5845, 0.6743, and 0.7258, respectively. (E-H) Dynamics of virus spread in different microenvironment in (A-D). Relative infected areas on the left half (blue, dotted), right (red, dotted) half, and whole (black, solid) tumor spheroid for four cases in Figures 4A-4D (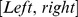 = 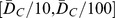 in (A), 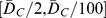 in (B), 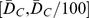 in (C), 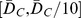 in (D)).

Figure 4 shows virus spread at 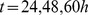 when a combination of different diffusion coefficients on left and right half of the square domain (second column in Figure 4). Patterns of Infected areas for different combinations show that virus spread is significantly inhibited in the area of low diffusivity. When diffusivity of Chase is fixed on the left half of the domain but decrease in the right half of the domain, most tumor cells on the left side were infected but not many cells near the center of the tumor mass on the right hand side were infected. For example, in Figure 4C, base value of diffusivity was assigned on the left half (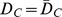) and 100-fold smaller values of diffusivity of Chase was assigned on the right half (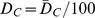). This difference in diffusivity leads to different patterns of virus infection on the left and right half of the domain. While virus infection is completed throughout the left half, only tumor cells on the periphery of the spheroid was infected in the right half of the domain. Similar patterns were observed in the case of 10-fold smaller 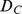 value on the right in Figure 4D (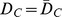 on the left; 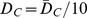 on the right). When the diffusivity is decreased on the left as well and 100-fold smaller diffusivity was fixed on the right (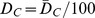), the infected area on the left also is decreased (Figures 4A-4B) as well. Figures 4E-4H show relative infected areas on the left, right, and whole domain, respectively, for the four cases in Figures 4A-4D. The simulation results in Figure 4 have implications for the efficacy of virus spread in the brain when chase components are injected a site near a tumor since brain tissue including white and gray matter is heterogeneous in nature.

### Testing hypothesis for real tumors *in vivo* and optimal solution of OV treatment

Muir *et al*. [51] were able to induce secretion of bacterial chondroitinase ABC from mammalian cells by modifying N-glycosylation sites. In this section, we assumed that infected tumor cells secrete the enzyme since the virus use the cell body to create this enzyme inside the infected cell. In this section, we use our model to test the results of treatment of tumor *in vivo* by genetically engineered virus that secrete ECM degradating enzyme through infected cells instead of injecting enzyme 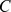, which acts passively outside the growing spheroid. In order to take into account the effect of secretion of enzyme by virus through infected cells we set in the equation (18)
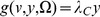
(24)where 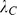 is the secretion rate of the enzyme from infected cells.

**Figure 5:**
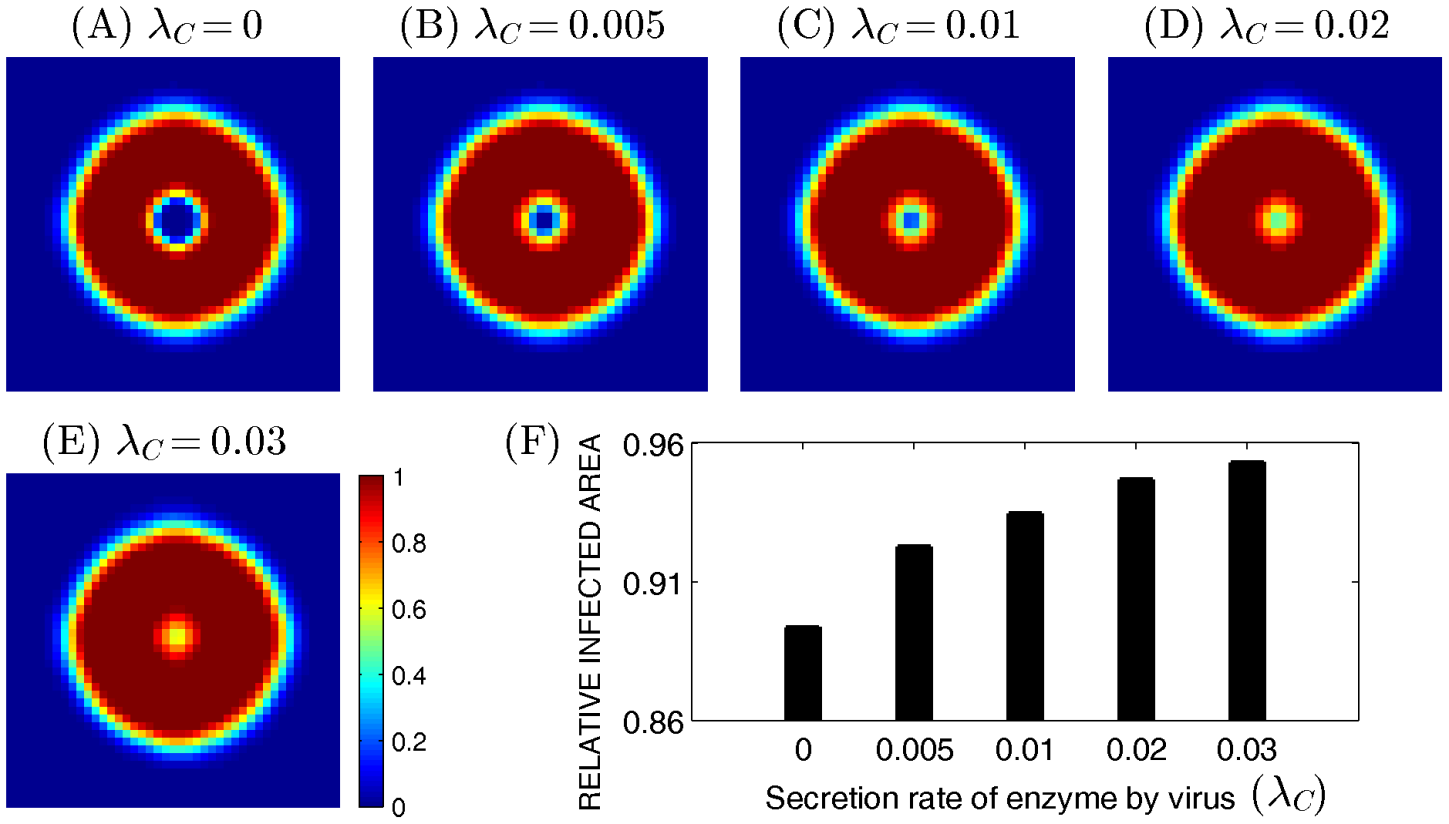
Effect of 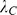 on virus infection. (A-E) The profiles of infected cells for various 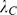 (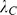 = 0 in (A), 0.0005 in (B), 0.01 in (C), 0.02 in (D), 0.03 in (E)) were shown at 60 hours postinfection. The enclosing box size is 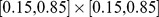. (F) Infected area at 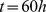 for various 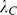 in (A-E). As 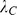 is increased, the infected area is increased.

Figure 5 shows profiles of infected tumor cells, at time 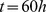, in response to various secretion rate of the enzyme by infected cells (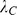 = 0, 0.005, 0.01, 0.02, 0.03). An increase in indirect secretion of the enzyme by infected cells effectively promotes penetration of the enzyme toward the tumor center and degradation of the ECM components: as 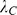 is increased, the infected area is increased. For example, while tumor cells at the center of tumor mass were not infected at 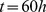 in the the control case, almost all tumor cells were infected even at the tumor center when 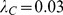.

**Figure 6:**
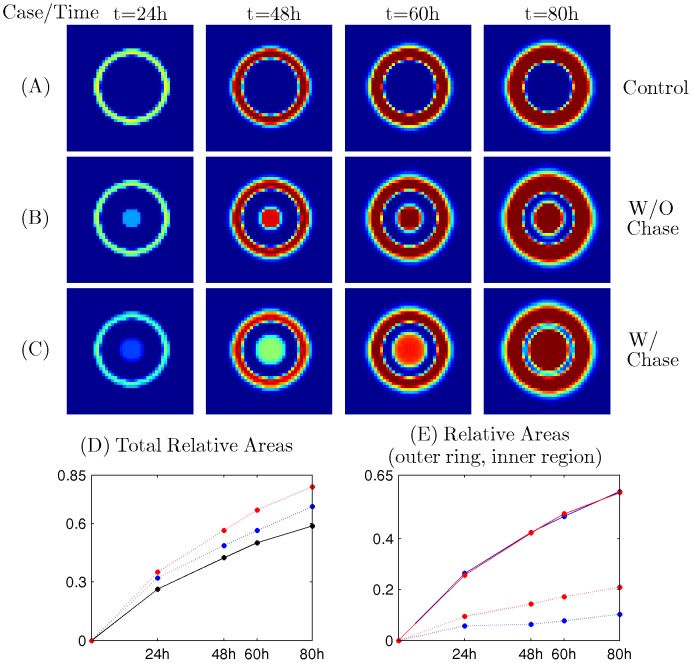
Effect of intratumoral injections of virus. Partial portion of virus (%15 of virus) was injected at the tumor center with (B)/without (C) Chase treatment and remaining %85 of virus was injected outside the tumor with Chase treatment at the tumor center For control case, same amount of total virus was injected outside the tumor. (A-C) Profiles of infected cells at t = 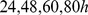 for control case (A), case without chase (B) and case with chase (C). 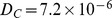 while other parameters are fixed as in Table 1. (D) Time course of total relative areas for the case of control (black solid; Figure 6A), without Chase (blue dotted; Figure 6B), and with Chase (red dotted; Figure 6C). Injection strategies on different locations improves the tumor infection relative the control. With treatment of Chase at the tumor center, relative infected area is even further improved. (E) Time course of relative areas of outer ring (solid) and inner region (dotted) for the case without Chase (blue, Figure 6B), and with Chase (red, Figure 6C).

We next consider a situation where be virus injection takes place at different locations. Figures 6A-6C show the efficacy of redistributed injection for the OV treatment with/without Chase-ABC relative the control case. We first inject the 15% of total OVs in the center of the growing glioma with/without treatment of Chase and inoculate the remaining OVs (85%) on the periphery of the tumor along with Chase treatment. For control case, same amount of total virus was injected outside the tumor. Profiles of infected cells at t = 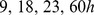 for control case (A), case without chase (B) and case with chase (C) were shown in Figures 6A-6C. While virus penetration begins on the periphery of the spheroid and stops in middle-way toward the center at final time (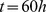) in the control case (Figure 6A), viral infection at the center of the spheroid spread outward aggressively in the case of viral injection at the center (Figures 6B-6C). In the case of Chase treatment at the injection site at the center of the spheroid (Figure 6C), viral infection is even more aggressive and almost all tumor cells were infected at final time (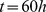). Figure 6D shows time courses of total relative areas for the case of control (black solid; Figure 6A), without Chase (blue dotted; Figure 6B), and with Chase (red dotted; Figure 6C). Injection strategies on different locations (red,blue) improves the tumor infection compared to the control (black solid). Treatment of Chase at the tumor center (red dotted) leads to even faster viral spread. Figure 6E shows time courses of relative areas of outer ring (solid) and inner region (dotted) for the case without Chase (blue, Figure 6B), and with Chase (red, Figure 6C). While infection areas of outer ring for both cases with/without Chase treatment are same at 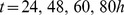 (blue solid, red solid), the case with Chase treatment (red dotted) at the center of tumor spheroid leads to faster spread of virus compared to the case without Chase (blue dotted). The injection amount of virus is limited due to safety concern to the patients from increased pressure inside the brain. Therefore, given MRI images of patient brain, we would like to optimize the efficacy of the treatment with limited number of virus per total injection, by carefully choosing injection sites. Our simulation results (Figure 6) which show improved efficacy by viral injections at both the center of the tumor and on its periphery, are somewhat supported by recent clinical data reported in Markert *et al*. [56]. Indeed, Markert *et al*. [56] investigated the efficacy of OV treatment in six human patients by two injections of OV. The first inoculation of 13% of 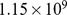 pfu of G207 in the center of the tumor mass was followed by a surgical resection of the tumor mass. The remaining virus (87%) was injected into brain tissue near the boundary of resected tumor area. In this study, virus was injected in tumor tissue by a catheter that was left in place, and the tumor resected en bloc 2–5 days later. Tissue was carefully divided into three sections a distal slice, a more proximal and the most proximal referred. Evaluation of HSV polymerase DNA in the tissue showed a decrease of 1 to two logs between the distal and proximal tissue in four of the six patients.

### Testing hypothesis on efficacy of a viral therapy on eradicating the invasive glioma cells

**Figure 7:**
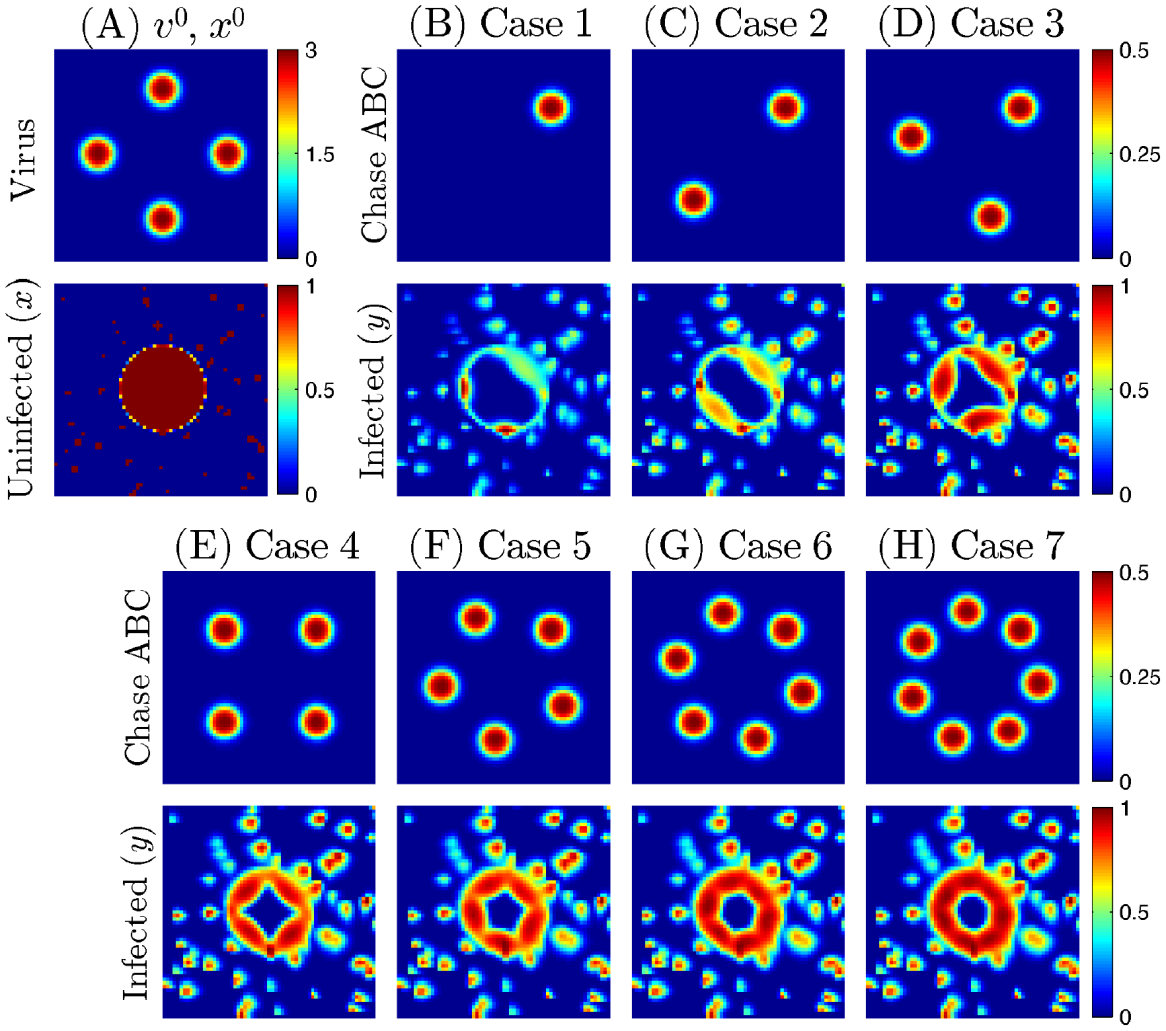
Effect of Chase-ABC injections on killing infiltrating glioma cells. (A) Initial distribution of virus (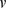) and tumor cells (invasive cells outside the tumor core and proliferative cells within the ellipsoidal tumor bulk; 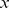). (B-H) Initial profiles of Chase-ABC (upper panels) and infected cell density at final time 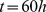 (lower panels). Viruses were injected on the periphery of the tumor bulk and Chase-ABC was injected at the different sites with equal strength. Number of injection sites: 1 (B), 2 (C), 3 (D), 4 (E), 5 (F), 6 (G), 7 (H). All parameters are fixed as in Table 1.

**Figure 8:**
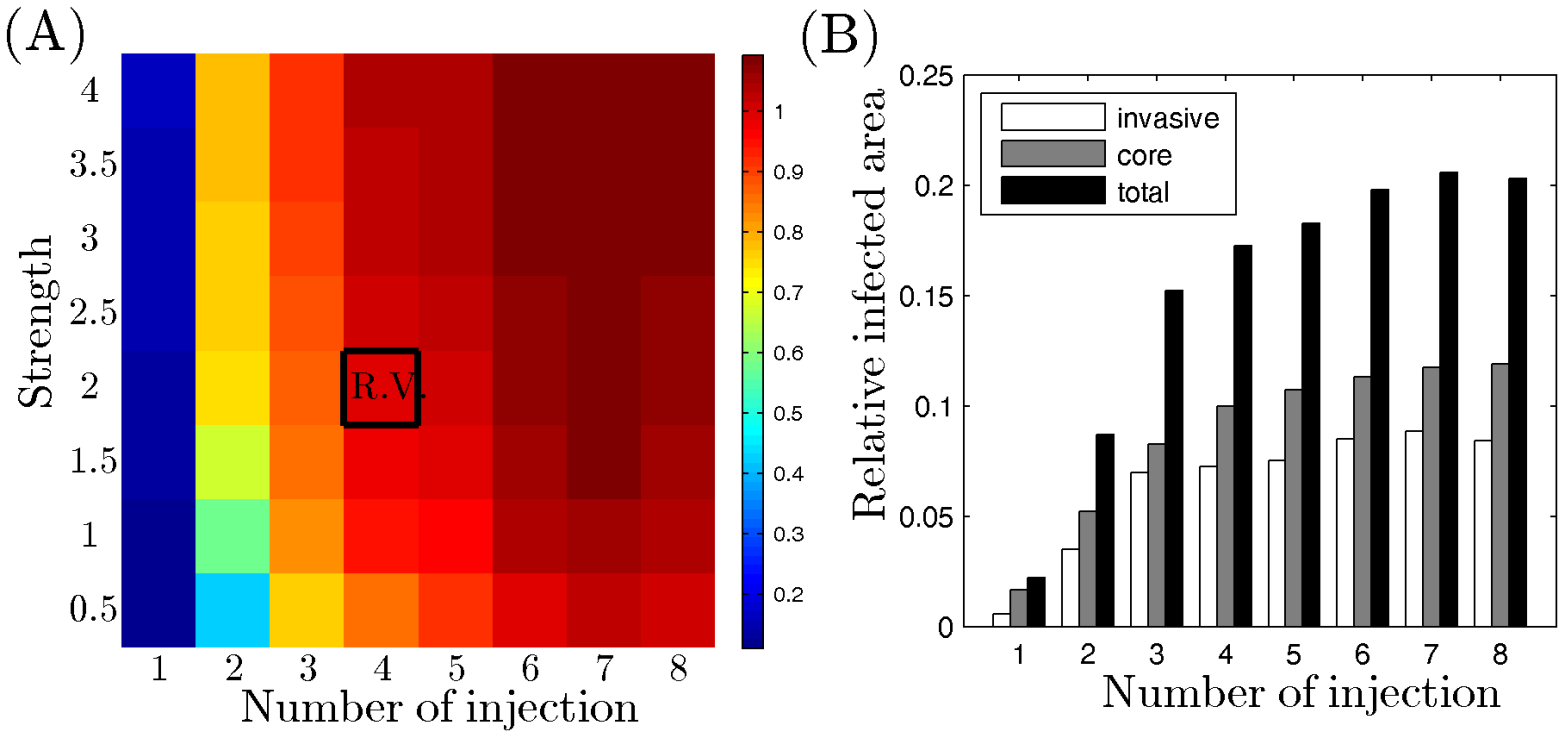
Optimal strategies of killing infiltrating glioma cells with various Chase-ABC injections. (A) Relative infected area for various number of sites and injection strength of the Chase-ABC. Viruses were injected on the periphery of the tumor bulk and the Chase-ABC was injected at the different sites with different strength. (B) Relative infected area for invasive (white), core (gray), and total glioma cells in eight cases in Figure 7. All parameters are fixed as in Table 1.

High invasiveness of glioma cells is the major challenge in treatment of glioma due to regrowth of the tumor after conventional treatment options such as surgery and chemo- and radio-therapy. Innovative therapeutic approaches of targeting these invasive cells are necessary in order to improve clinical outcome [57]. Here we test some hypotheses on the efficacy of a possible viral therapy on eradicating invasive glioma cells that shed from the main tumor core. For this purpose, we assume that the Chase-ABC is delivered by injection at different sites (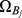), so that
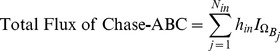
(25)where 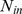 is the number of injection sites and 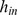 is the injection strength at 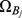. For simplicity, we assume an initial configuration of a spherical tumor in the brain tissue with invasive cells at random locations outside of the tumor core as in Figure 7A. The injection sites were assumed to be located on the periphery of the spherical tumor. Figures 7B-7H show the density of the infected tumor cells for injections of Chase-ABC at different sites, with the same strength at each site after 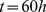. In Figure 7B the Chase-ABC is injected near the north east (NE) corner, resulting in more virus-infected area (*i.e.*, more virus-infected tumor cells) in the NE area than elsewhere. On the other hand, since the extracellular virus do not move freely in the south west (SW) corner (where no Chase-ABC was injected), the virus are more localized, resulting in the higher concentration of infected tumor cells, but in very narrow strips. Figures 7B-7H show that by strategically injecting increasing number of Chase-ABC, the number of infected cells increases, not only inside the tumor but also among the invasive cells We note however that very little improvement occurs after injections at more than four sites. This is so because the Chase-ABC at the four sites manages to diffuse quite effectively in the entire region after 60 hours, and, furthermore, the effectiveness of Chase-ABC in the removal of CSPG reaches saturation level 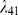 as the concentration of Chase-ABC increases to infinity (see equation (8)). We conclude that for a tumor as in Figure 7A, it is not necessary to inject Chase-ABC in more than four strategically chosen sites. Larger tumors, or tumors with more invasive cells distribution may require additional injection sites. The relative infected areas for invasive (white), core (gray), and total glioma cells in seven cases are shown in Figure 8B. As the number of injection sites is increased, the infected population of both invasive and proliferating tumor cells inside the core is increased overall. The model predicts high improvement on the efficacy of the treatment when 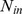 is increased from 1 to 4 (white bar in Figure 8B). However, the efficacy of the treatment on infecting the infiltrating glioma cells is not significantly improved if 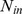 is further increased (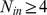) due to the saturation phenomenon mentioned above. Figure 8A shows the relative infected area of both infiltrating and proliferative tumor cells for various number of injection sites (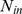) and injection strength (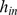). The efficacy is increased as 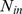 is increased for fixed 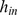 and as 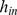 is increased for fixed 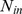. However, because of saturation effect, there is no significant increase in the total infected area if 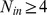 or if 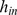 is increased beyond a certain limit. Since too many injections or high concentrations of Chase-ABC may harm normal cells, our model suggests that the choice of a small number of strategically located sites (e.g. four) with limited concentration of Chase-ABC is the optimal strategy that would be effective in eradicating tumor cells not only in the visible core tumor but also in the isolated cluster as long as the cluster of invasive cells are not too far from the main tumor bulk.

**Figure 9:**
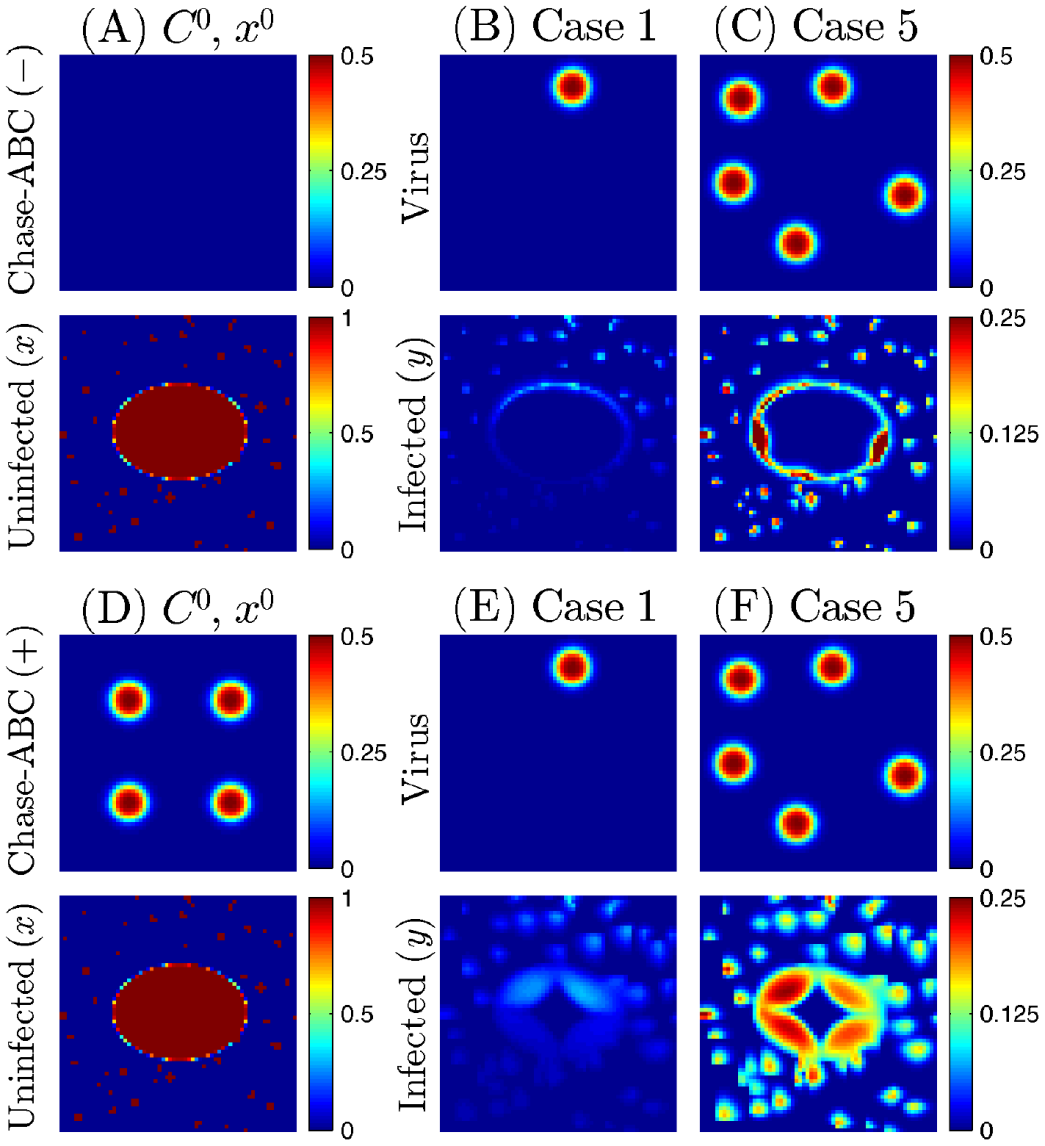
Effect of intravenous injection of viruses on killing infiltrating glioma cells with no Chase-ABC(−) and fixed Chase-ABC(+) supply. (A,D) Initial distribution of Chase-ABC (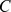) and tumor cells (invasive cells outside the tumor core and proliferative cells within the ellipsoidal tumor bulk; 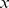). (B,C) Initial profiles of virus (upper panels) and infected cell density at final time 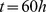 (lower panels) in the absence of Chase-ABC when viruses were supplied through one (B) and five (C) blood vessel sites with equal strength after intravenous injection. (E,F) Same as (B,C) but in the presence of Chase-ABC at four sites in the upper panel in (D). All parameters are fixed as in Table 1.

Virus could also be transported to brain tumors through the vascular route [58]. We test the efficacy of Chase-ABC on killing both infiltrating cells and tumor bulk when virus supply is through intravenous injections at different sites (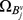). Thus we have the following virus supply via the intravenous injection:
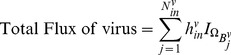
(26)where 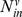 is the number of blood sites for virus transport and 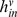 is the strength (or concentration) of the viruses at 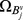. Invasive tumor cells were initially distributed at random locations outside the tumor bulk (lower panel in Figures 9A,9D). In the absence of Chase-ABC, viral infection are not effective due to limited virus spread for both invasive cells and proliferating cells in the main tumor bulk when viruses were supplied through one (Figure 9B) and five (Figure 9C) blood vessel sites. The infection rate for infiltrating cells is increased as 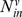 is increased (Figure 9B 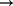 Figure 9C). However, there is no significant improvement in overall penetration of the main tumor bulk due to lack of Chase-ABC. On the other hand, there is a significant improvement in infecting the bulk tumor when Chase-ABC was injected at four sites (Figures 9E,9F). Upper panels in Figures 9B,9C,9E,9F show the initial distribution of viruses. In Figure 9B a higher viral infection is also observed in the upper part of the domain in the presence of Chase-ABC due to the location of the virus injection site. Thus, presence of more blood vessels close to the invasive glioma cells and bulk tumor naturally increases the efficacy of killing infiltrating tumor cells by viral infection (Figure 9E 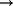 Figure 9F). As in the previous test above, the larger number of blood sites as a source of viral injection (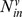) does not significantly improve the efficacy of viral spread and infection for both invasive cells and the bulk tumor (not shown here). Too large numbers of viruses may harm normal cells and there exist a limit for virus injection. Especially in the case of intravenous injections, control over how much viruses can be transported is rather limited due to the different microenvironment that the tumor resides in. In brain, entry of even small biological agents into brain tissue is usually more complex than other organs because of the blood-brain barrier (BBB) [59], [60]. Despite successful transport of virus via blood routes and viral infection of gliomas (VSVrp30a; [58]), the delivery of viruses may depend on many factors such as biochemical conditions of blood vessels, tissue composition and geometry. Therefore, given blood distributions of the patients, one has to use optimized patient-specific injection strategies with minimal side effects, i.e., without killing normal healthy brain cells, in order to eradicate both invasive cells and the tumor bulk.

### Effect of virus injection with Chase-ABC on killing primary and secondary tumor cells

The experimental results reported in [11] were obtained only in the case of one primary tumor. However glioma cells, being highly invasive, are more likely to have infiltrated into the brain tissue and formed multifocal or satellite lesions at time of diagnosis. In a recent univariate analysis of 15 imaging features of patients with GBM [61], Pope *et al*. found that multifocality/satellite lesions, in addition to the noncontrast-enhancing tumor and edema, were statistically significant prognostic indicators. It is therefore interesting to determine the outcome of applying viral therapy in the case where primary and secondary tumors are present. This is particularly challenging in the case where as the size of one of the secondary tumor is increasingly small (representing small satellite lesions) or when the two tumors are not too close together or not too far apart. In this section we consider these two situations.

**Figure 10:**
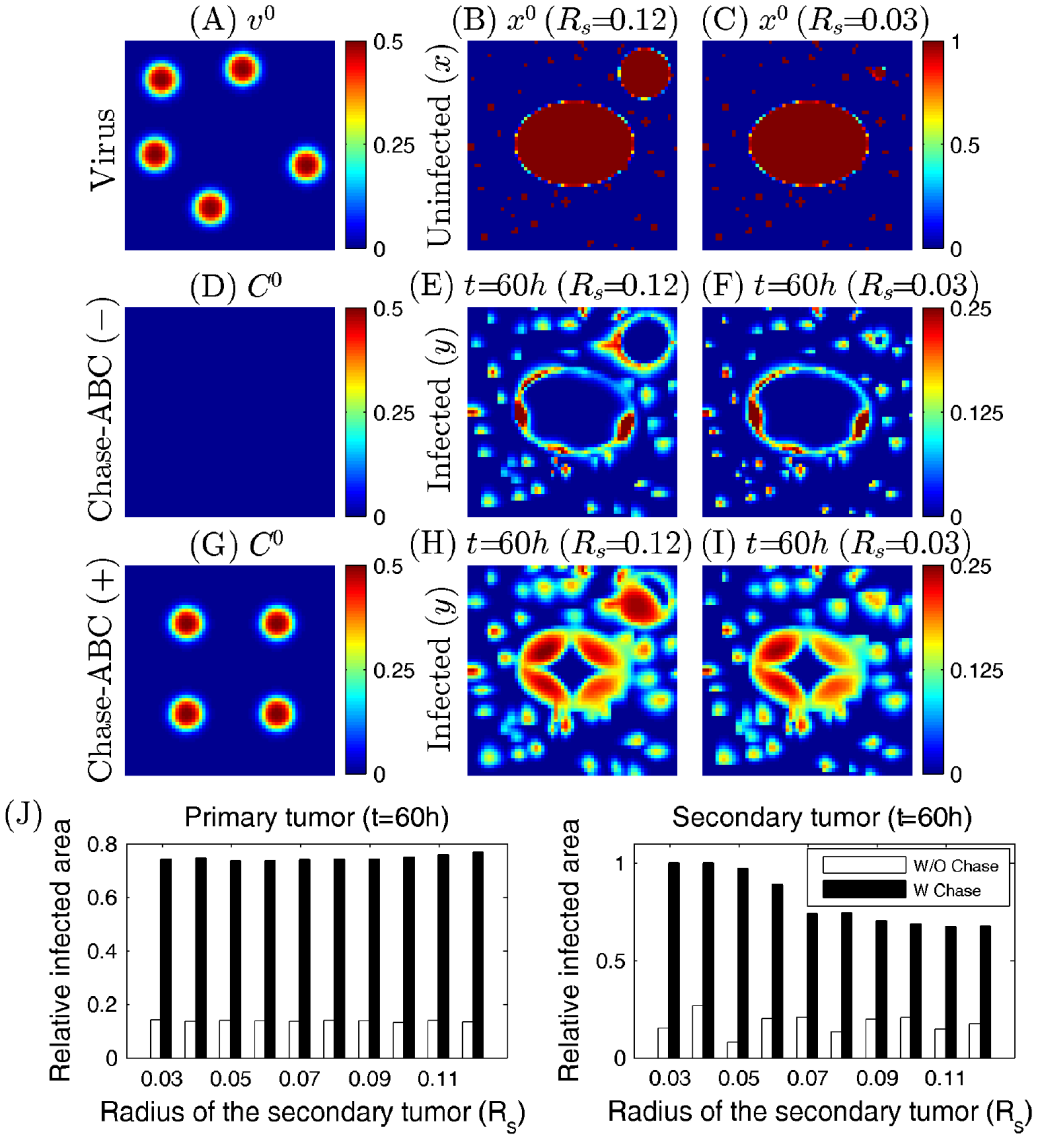
Effect of intravenous injection of viruses on killing primary and secondary tumor cells with no Chase-ABC(−) and fixed Chase-ABC(+) supply. (A) Initial distribution of viruses (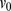) (B,C) Initial profiles of uninfected tumor cells (primary tumor with a ellipsoidal shape and spherical secondary tumor as well as invasive cells outside these tumors; 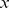) when the radius of the secondary tumor (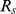) is relatively large (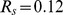 in (B)) and small (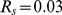 in (C)). (D,G) Initial profiles of Chase-ABC (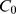) in the absence of Chase (Chase-ABC(−) in (D)) and presence of Chase-ABC (Chase-ABC(+) in (G)). (E,F) Infected cell densities at final time 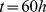 for a large (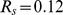; (E)) and small (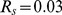; (F)) secondary tumor size in the absence of Chase-ABC. (H,I) Same as (E,F) but in the presence of Chase-ABC. (J) Relative infected areas of the primary (left) and secondary (right) tumors for various radius of the secondary tumor (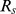 = 0.03, 0.04, 0.05, 0.07, 0.08, 0.09, 0.1, 0.11, 0.12) in the absence of Chase (white bar) and presence (black bar) of Chase-ABC. All parameters are fixed as in Table 1.

We first investigate the effect of the secondary tumor size on virus infection of primary and secondary tumors in the absence and presence of Chase-ABC. Figures 10B,C show initial distributions of uninfected tumor cells (primary tumor with a ellipsoidal shape and spherical secondary tumor as well as invasive cells outside these tumors) when the radius of the secondary tumor (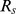) is relatively large (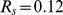; Figure 10B) and small (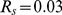; Figure 10C). Viruses (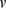) were injected at five sites outside the primary and secondary tumors (Figure 10A). In the absence of Chase (Chase-ABC(−)), there are no much differences in relative infection rates of both primary and secondary tumors for the large (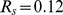; Figure 10E) and small (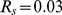; Figure 10F) size of the secondary tumor. In fact, relative infected areas does not change much for various radii (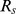 = 0.03, 0.04, 0.05, 0.07, 0.08, 0.09, 0.1, 0.11, 0.12) of the secondary tumor in both primary (left panel; white bar in Figure 10J) and secondary (right panel; white bar in Figure 10J) tumors in the Chase-ABC(−) case. In the presence of Chase-ABC (Chase-ABC(+)), there are significant improvements relative to the Chase-ABC(−)) case in infection rates of both primary (left panel; black bar in Figure 10J) and secondary (right panel; black bar in Figure 10J) tumors for the fixed 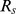. As the secondary tumor size (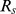) is increased, the relative infected area of the secondary tumor is decreased but the infection rate of the primary tumor stays almost constant. For example, while there are no much differences in infected areas of the primary tumor at final time 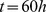 for a large (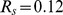; Figure 10H) and small (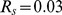 in Figure 10I) secondary tumor, a large portion of the larger secondary tumor was not infected (upper right corner in Figure 10H) and all areas of the smaller secondary tumor were infected (upper right corner in Figure 10I). From the above experiments we conclude that with Chase treatment, the relative infected area increases as the size of the secondary tumor decreases. This is due to efficient spread of Chase throughout the relatively smaller satellite, followed up by the aggressive and effective degradation of CSPG, and thus resulting in much less physical resistance for the virus spread.

**Figure 11:**
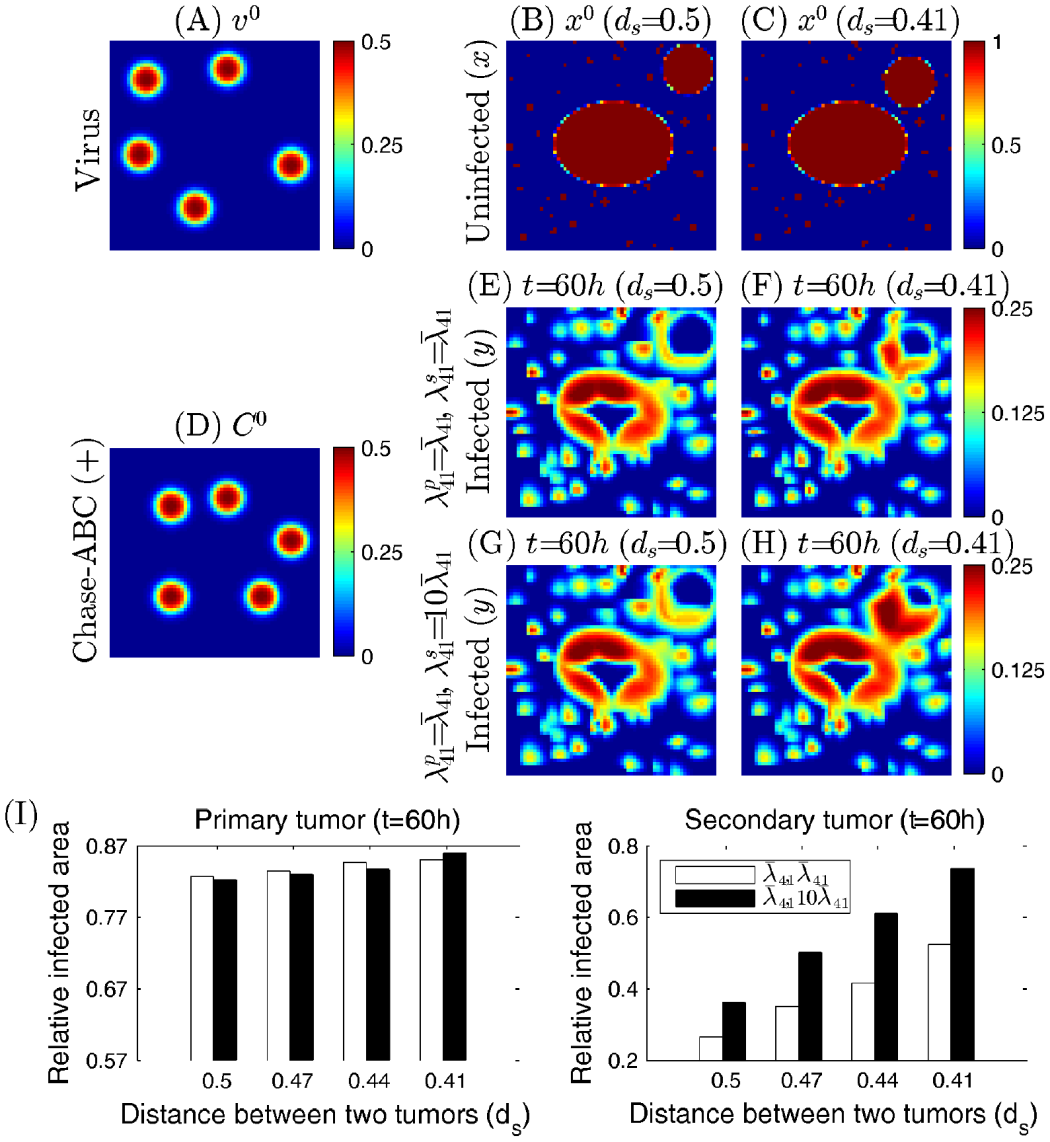
Effect of oncolytic therapy on killing primary and secondary tumor cells with different biomechanical properties. (A) Initial distribution of viruses (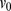) (B,C) Initial profiles of uninfected tumor cells (primary tumor with a ellipsoidal shape and spherical secondary tumor as well as invasive cells outside these tumors; 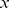) when the distance between primary and secondary tumors (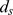) is relatively large (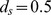 in (B)) and small (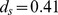 in (B)). (D) An initial profile of Chase-ABC (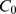) (Chase-ABC(+)). (E,F) The infected cell density at final time 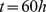 for the large (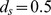 in (E)) and small (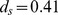 in (F)) distances for the control case (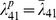 (primary tumor), 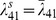 (secondary tumor)). (G,H) Same as (E,F) but for the enhanced ECM-degradation strength of the secondary tumor (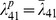, 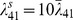). (I) Relative infected areas of the primary (left panel) and secondary (right panel) tumors for various distances between two tumors (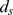 = 0.5, 0.47, 0.44, 0.41). All other parameters are fixed as in Table 1.

As a second test, we investigate the effect of the distance between primary and secondary tumors on virus infection of these tumors in the presence of Chase-ABC (Chase-ABC(+)). Figures 11B,C show initial distributions of uninfected tumor cells (primary tumor with a ellipsoidal shape and spherical secondary tumor as well as invasive cells outside these tumors; 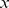) when the distance between the primary and the secondary tumors (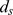) is relatively large (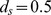; Figure 11B) and small (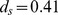; Figure 11C). Here, the distance between two tumors was defined as the distance between the center points of these tumors, *i.e.*, 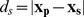 where 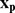 and 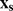 are the center points of the primary and the secondary tumors, respectively. Viruses (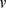) were injected at five sites outside the primary and secondary tumors (Figure 11A). Initial profile of Chase-ABC (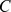) is shown in Figure 11D. In order to see the effect of Chase-driven enhancement of ECM degradation on the efficacy of virus spread on both tumors, two different ECM degrading rates (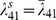 for the control (normal) case; 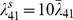 for the high degradation rate) for the secondary tumor were considered while the rate for the primary tumor was fixed (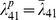). Here, 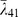 is the control parameter in the model. Figure 11I shows relative infected areas of the primary and secondary tumors for various 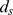 (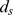 = 0.5, 0.47, 0.44, 0.41). In both cases, relative infected areas for both primary and secondary tumors are increased as the distance between these tumors (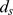) is decreased (white bars in Figure 11). When the ECM-degrading strength of the secondary tumor is increased by 10-fold (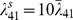), the relative infected areas of the secondary tumor is significantly increased regardless of distance between two tumors (right panel in Figure 11I). However, the model predicts that the virus infection of the primary tumor in response to the larger 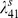 depends on 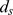 in a non-linear fashion (left panel in Figure 11I). While the enhanced strength of ECM-degradation of the secondary tumor (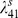) slightly decreases the relative infected area of the primary tumor for a relatively larger fixed 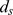 (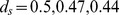), it promotes virus spread in the primary tumor for the close secondary tumor (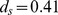). This is because the relative spread of Chase toward the primary tumor is reduced due to increased degradation of the secondary tumor followed by the increased spread speed of Chase-ABC toward the secondary tumor. When the secondary tumor is close to the primary tumor (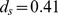), the high viral activity in the secondary tumor may induce the fast viral spread to the neighboring primary tumor leading to increased viral infection at the primary tumor site. For example, one can see the broad infected areas in the secondary tumor and bridge-like spread of viruses between the primary and secondary tumors when these tumors are close each other (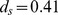; Figure 11H) while the limited spread of viruses is observed in the remotely located primary tumor (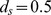; Figure 11G). Invasive cells are more susceptible to virus infection due to free access of viruses and these infiltrating cells in the gap between the two tumors also contribute to the larger infection area of the primary tumor by accelerating the generation of available viruses in the neighboring region close to the primary tumor, acting as a bridge for virus transport between two tumors. Control cases for these remote and close satellites are shown in Figures 11E,11F, respectively. From the above experiments we see with Chase treatment, the relative infected area increases as the distance from the satellite to the primary tumor decreases. This is due to the advantage of having larger virus population near the primary lesion from the active viral infection, and hence heavy viral duplication, on the outer rim of the primary tumor.

Combining the above two experiments it follows that the relative infected area will be relatively large if the secondary tumor is small and close to the primary tumor; this suggests that Chase may perhaps be considered as anti metastatic enhancement of viral therapy.

## Discussion

Genetically manipulated oncolytic viruses showed great potential of destroying cancer cells with quick replication ability but with minimal damage to normal healthy cells [4]. Despite its great potential and popularity in China, US, and Europe [5], its inefficient OV spread over the entire tumor ECM has been recognized as a major challenge in anti-tumor efficacy [5], [62].

In gliomas, systemic metastasis is rare and 90% of gliomas recur locally (less than a few 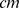 from the resected area) [63], [64], making gliomas good targets of virus therapy [56]. Accumulated CSPGs in the glioma ECM is associated with tumor growth/invasion and angiogenesis [29] by increasing tortuosity of the extracellular space and interstitial pressure within the tumor [11], resulting in blocking transport of large therapeutic agents [22]. Tumor proliferation and dispersion can be inhibited when ECM macromolecules are reduced [30], [31]. Dmitrieva *et al*. [11] found that OV spread was significantly enhanced by OV Chase treatment, making ChaseABC one of important therapeutic molecules that can improve interstitial transport of therapeutic agents into the tumor. In this study, Chase expression did not interfere with viral cytotoxicity. Virus penetration in a local microenvironment may depend on many factors such as ECM composition and deposition of collagen and fibrinogen from highly permeable angiogenic vessels [23], and constant remodeling of ECM [65].

In this paper we model the phenomena of Chase-assisted virus spread using a system of partial differential equations. The model variables include tumor cells (infected or uninfected by virus), dead tumor cells, OV, Chondroitinase, and the ECM component. The model simulation are in qualitative agreement with the experimental results of Dmitrieva *et al*. [11]. It is a common practice to inject the virus into several different location within or outside the tumor mass. The mathematical model can be used to suggest testable hypotheses on preferred locations for such injections. In the absence of Chase, viral spread is very limited after injection of the oncolytic virus and only the cells on the periphery of the spheroid are infected leaving bulk of tumor cells uninfected at the center of tumor mass. On the other hand, viral penetration toward the center of tumor spheroid is improved with assistance of Chondroitinase Chase and much larger portion of tumor cells are infected near the center of the tumor spheroid. This improvement is due to the ability of Chase to degrade the thick network of ECM allowing virus to enter more easily into the neighboring site. This viral penetration can be improved when smarter virus is used, *i.e.*, when virus is equipped with an ECM degrading enzyme through infected cells [51]. This effect was tested in our model and its improved efficacy was illustrated in our model simulations (Figures 2). Therefore, development of smart virus armed with bacterial chondroitinase ABC or other powerful ECM degrading enzyme may provide a pivotal way of promoting virus spread within the tumor by increasing enzyme activity, thus killing off tumor cells. However, we note that the use of these degrading enzymes may cause serious complications such as hemorrhagic necrosis of brain, neurodegenerative diseases [26], and optic glioma growth [27]. Treatment failures in glioblastoma are partially attributed to cellular heterogeneity [66], [67] and local microenvironment [36]. We also found that the microenvironment plays a significant role in viral spread as demonstrated in Figure 4, and this has implication for brain tissue with variable 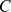-diffusion coefficient 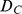. Our model predicts that the virus spread is quite selective in given microenvironment they are imposed in and location-specific delivery of virus is needed in order to widen infected area and improve the efficacy of the virus treatment.

Infiltrating glioma cells create major challenge in glioma treatment due to their invisibility for standard medical devices such as MRI [68]. These cells may infiltrate the narrow intercellular space between normal cells in the brain tissue by deforming cell body and nucleus with effective use of myosin II motor [69]. Therefore, it is important to develop an efficient delivery method to target and kill dispersed tumor cells [58]. In orthotopic glioma models in rodent brain, systemic application of intravenous vesicular stomatitis virus (VSV) may be effective on selective targeting and infecting remote satellite glioma cell clusters as well as the tumor bulk [58], [70]. We tested the efficacy of virus therapy on eradicating those cells in addition to killing the tumor cells in the main tumor mass. Under the biochemical and biophysical constraints, optimal virus therapy with Chase-ABC treatment would be also effective on killing these invasive cells as long as they are staying within the local areas. Our study found that the injection of Chase-ABC in addition to injection of virus will be effective not only in infecting and killing the tumor bulk but also in eradicating infiltrating glioma cells even in the presence of the low density of blood vessels. However, this may not be effective on killing these migratory glioma cells in the far field even with the optimal strategies [46]. In addition, entry of even small biological agents into brain tissue is usually more complex than other organs because of the blood-brain barrier (BBB) [59], [60], [71], adding more obstacles in viral therapy. Some of replicating therapeutic viruses have not been able to disseminate to some parts of tumor [72], [73]. Viral therapy may affect only local areas near blood vessels [73]. For instance, a histological analysis from transduced human glioma showed low transduction efficiencies with a retrovirus LacZ marker gene and adenoviral LacZ marker gene in some regions of the tumors while the neighboring areas showed much lower efficiency. These studies indicate that multiple injections are needed to infect fully the tumor bulk or the walls of the tumor cavity [74]–[76]. Some improved viruses were shown to target and kill cells in different regions inside the tumor via vessel leakage, *i.e.*, virus seeding at multiple sites [58]. Our study predicts the improvement in an intravenous viral delivery when appropriate levels of Chase-ABC were used in an optimal way. Some viruses also depend on specific genetic defects such as RAS or myc oncogene activity, cell cycle status, p53 status, or hypoxic environment [77]–[79]. Therefore, optimized patient-specific strategies would be necessary in order to get better clinical outcomes, without causing catastrophic results. From our *in silico* experiments, we found that the size of tumor satellites and distance between the primary tumor and secondary ones may determine the efficacy of viral therapy with Chase treatment: (i) the relative infected area increases as the size of the secondary tumor decreases because of effective degradation of CSPG and reduction in physical resistance of the viral spread. (ii) the relative infected area increases as the distance from the satellites to the primary tumor decreases because of the advantage of having relatively large virus population near the primary lesion from the active viral infection, and hence heavy viral duplication, on the outer rim of the primary tumor.

In the current model, we did not take into account many factors such as cell migration [1], angiogenesis, stromal cells within the tumor [80], constant remodeling of ECM from blood flow, immune system control or growth factors [81] such as epidermal growth factors (EGF) [82] and fibroblast growth factors (FGF) [83] that may play significant roles in regulating efficacy of oncolytic virus therapy. In fact, those growth factors were used as a tag or associated molecule for targeting cancer cells by oncolytic virus [84]. For example, Verheije *et al*. [85] showed that infection of malignant human glioblastoma U87
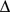
EGFR cells resulted in release of progeny virus and efficient elimination of cancer cells *in vitro* [85]. Many research groups studied strategies for targeting of polymer-coated adenovirus to the EGFR [86]. How temporal-spatial dynamics of these growth factors, such as fluctuations, affect glioma cell invasion/proliferation or efficacy of viral therapy is largely unknown. Also, despite all advantages of oncolytic virus as carrier for gene therapy or themselves, the clinical application is limited due to practical safety issues such as immune response [87] and tropism of virus [88], and rather systemic administration is necessary [84], [88]. Also, we might get better insight by considering both free (interstitial) and bound virus [89] instead of one population of virus. However, the mathematical model developed herein is a first step toward incorporating these factors, which we hope to address in future work. We finally note that other approaches such as a hybrid model [55], [90], [91] can be useful to describe detailed mechanical effects such as virus penetration of a cell, biomechanical control for duplication of virus within a cell and intracellular signals within a cell. It is also not clear how effectively virus therapy would work for small group of invisible migratory cells which has spread over other brain tissue, are free from surgery due to low density, and eventually recur [1]. Individual cell migration might depend on many factors such as chemotaxis, haptotaxis, cell-cell adhesion [36] and other microenvironmental factors.

## Supporting Information

Text S1 **Detailed description of the models and parameters used.**
(PDF)

## References

[1] Kim Y, Roh S, Lawler S, Friedman A (2011) miR451 and AMPK/MARK mutual antagonism in glioma cells migration and proliferation. PLoS One 6: e28293. 22205943 10.1371/journal.pone.0028293 PMC3243681

[2] Jacobs V, Valdes P, Hickey W, Leo JD (2011) Current review of in vivo GBM rodent models: emphasis on the CNS-1 tumour model. ASN NEURO 3: e00063. 21740400 10.1042/AN20110014 PMC3153964

[3] Barcellos-Hoff M, Newcomb E, Zagzag D, Narayana A (2009) Therapeutic targets in malignant glioblastoma microenvironment. Semin Radiat Oncol 19: 163–70. 19464631 10.1016/j.semradonc.2009.02.004 PMC3538148

[4] Msaouel P, Dispenzieri A, Galanis E (2009) Clinical testing of engineered oncolytic measles virus strains in the treatment of cancer: an overview. Curr Opin Mol Ther 11: 43–53. 19169959 PMC2717625

[5] Kaur B, Cripe T, Chiocca E (2009) Buy one get one free: armed viruses for the treatment of cancer cells and their microenvironment. Curr Gene Ther 9: 341–355. 19860649 10.2174/156652309789753329 PMC2802461

[6] Kuriyama N, Kuriyama H, Julin C, Lamborn K, Israel M (2000) Pretreatment with protease is a useful experimental strategy for enhancing adenovirus-mediated cancer gene therapy. Hum Gene Ther 11: 2219–2230. 11084679 10.1089/104303400750035744

[7] Kim J, Lee Y, Kim H, Huang J, Yoon A, et al (2006) Relaxin expression from tumor targeting adenoviruses and its intra tumoral spread, apoptosis induction, and efficacy. Gene Ther 98: 1482–1493. 10.1093/jnci/djj397 17047197

[8] Haseley A, Alvarez-Breckenridge C, Chaudhury AR, Kaur B (2009) Advances in oncolytic virus therapy for glioma. Recent patents on CNS drug discovery 4: 1. 19149710 10.2174/157488909787002573 PMC2720101

[9] Hong C, Fellows W, Niranian A, Alber S, Watkins S, et al (2010) Ectopic matrix metalloproteinase-9 expression in human brain tumor cells enhances oncolytic hsv vector infection. Gene Ther 17: 1200–1205. 20463757 10.1038/gt.2010.66 PMC3228315

[10] Choi I, Lee Y, Yoo J, Yoon A, Kim H, et al (2010) Effect of decorin on overcoming the extracellular matrix barrier for oncolytic virotherapy. Gene therapy 17: 190–201. 19907500 10.1038/gt.2009.142

[11] Dmitrieva N, Yu L, Viapiano M, Cripe T, Chiocca E, et al (2011) Choindroitinase ABC I-mediated enhancement of oncolytic virus spread and antitumor efficacy. Clin Cancer Res 17: 1362–72. 21177410 10.1158/1078-0432.CCR-10-2213 PMC3140790

[12] Haseley A, Boone S, Wojton J, Yu L, Yoo JY, et al (2012) Extracellular matrix protein ccn1 limits oncolytic efficacy in glioma. Cancer research 72: 1353–1362. 22282654 10.1158/0008-5472.CAN-11-2526 PMC3366191

[13] Viapiano M, Matthews R (2006) From barriers to bridges: chondroitin sulfate proteoglycans in neuropathology. Trends Mol Med 12: 488–496. 16962376 10.1016/j.molmed.2006.08.007

[14] Dwyer CA, Matthews RT (2011) The neural extracellular matrix, cell adhesion molecules and proteolysis in glioma invasion and tumorigenicity. In: Garam M, editor, Molecular Targets of CNS Tumors, InTech. pp. 239–264.

[15] Wang C, Tong X, Yang F (2014) Bioengineered 3D brain tumor model to elucidate the effects of matrix stiffness on glioblastoma cell behavior using PEG-based hydrogels. Molecular pharmaceutics. 10.1021/mp5000828 24712441

[16] Ananthanarayanan B, Kim Y, Kumar S (2011) Elucidating the mechanobiology of malignant brain tumors using a brain matrix-mimetic hyaluronic acid hydrogel platform. Biomaterials 32: 7913–7923. 21820737 10.1016/j.biomaterials.2011.07.005 PMC3159794

[17] Delpech B, Maingonnat C, Girard N, Chauzy C, Olivier A, et al (1993) Hyaluronan and hyaluronectin in the extracellular matrix of human brain tumour stroma. European Journal of Cancer 29: 1012–1017. 10.1016/s0959-8049(05)80214-x 7684596

[18] Patan S (2004) Vasculogenesis and angiogenesis. In: Kirsch M, Black PM, editors, Angiognesis in Brain Tumors, Kluwer Academic Publishers. pp. 3–32.

[19] Klekner A (2013) Brain tumor invasion and angiogenesis. In: Lichtor T, editor, Evolution of the Molecular Biology of Brain Tumors and the Therapeutic Implications, InTech. pp. 3–36.

[20] Zimmermann D, Dours-Zimmermann M (2008) Extracellular matrix of the central nervous system: from neglect to challenge. Histochem Cell Biol 130: 635–53. 18696101 10.1007/s00418-008-0485-9

[21] Ramanujan S, Pluen A, McKee T, Brown E, Boucher Y, et al (2002) Diffusion and convection in collagen gels: implications for transport in the tumor interstitium. Biophys J 83: 1650–60. 12202388 10.1016/S0006-3495(02)73933-7 PMC1302261

[22] Moon L, Asher R, Fawcett J (2003) Limited growth of severed cns axons after treatment of adult rat brain with hyaluronidase. J Neurosci Res 71: 23–37. 12478611 10.1002/jnr.10449

[23] Netti P, Berk D, Swartz M, Grodzinsky A, Jain R (2000) Role of extracellular matrix assembly in interstitial transport in solid tumors. Cancer Res 60: 2497–2503. 10811131

[24] Eikenes L, Bruland O, Brekken C, Cde LD (2004) Collagenase increases the transcapillary pressure gradient and improves the uptake and distribution of monoclonal antibodies in human osteosarcoma xenografts. Cancer Res 64: 4768–73. 15256445 10.1158/0008-5472.CAN-03-1472

[25] Guedan S, Rojas J, Gros A, Mercade E, Cascallo M, et al (2010) Hyaluronidase expression by an oncolytic adenovirus enhances its intratumoral spread and suppresses tumor growth. Mol Ther 18: 1275–1283. 20442708 10.1038/mt.2010.79 PMC2911260

[26] Wang Y, Luo W, Reiser G (2008) Trypsin and trypsin-like proteases in the brain: proteolysis and cellular functions. Cell Mol Life Sci 65: 237–252. 17965832 10.1007/s00018-007-7288-3 PMC11131809

[27] Daginakatte G, Gutmann D (2007) Neurofibromatosis-1 (Nf1) heterozygous brain microglia elaborate paracrine factors that promote Nf1-deficient astrocyte and glioma growth. Hum Mol Genet 16: 1098–1112. 17400655 10.1093/hmg/ddm059

[28] Lin R, Kwok J, Crespo D, Fawcett J (2008) Chondroitinase abc has a long-lasting effect on chondroitin sulphate glycosaminoglycan content in the injured rat brain. J Neurochem 104: 400–408. 18005340 10.1111/j.1471-4159.2007.05066.x

[29] Viapiano M, Lawler S (2009) Glioma Invasion: Mechanisims and Therapeutic Challenges. Humana Press.

[30] Arslan F, Bosserhoff A, Nickl-Jockschat T, Doerfelt A, Bogdahn U, et al (2007) The role of versican isoforms v0/v1 in glioma migration mediated by transforming growth factor-beta2. Br J Cancer 96: 1560–8. 17453002 10.1038/sj.bjc.6603766 PMC2359935

[31] Viapiano M, Hockfield S, Matthews R (2008) Behab/brevican requires adamts-mediated proteolytic cleavage to promote glioma invasion. J Neurooncol 88: 261–272. 18398576 10.1007/s11060-008-9575-8 PMC3896091

[32] Grumet M, Friedlander D, Sakurai T (1996) Functions of brain chondroitin sulfate proteoglycans during developments: interactions with adhesion molecules. Perspect Dev Neurobiol 3: 319–330. 9117263

[33] Mok W, Boucher Y, Jain R (2007) Matrix metalloproteinases-1 and -8 improve the distribution and efficacy of an oncolytic virus. Cancer Res 67: 10664–10668. 18006807 10.1158/0008-5472.CAN-07-3107

[34] Bruckner G, Bringmann A, Hartig W, Koppe G, Delpech B, et al (1998) Acute and long-lasting changes in extracellular-matrix chondroitin-sulphate proteoglycans induced by injection of chondroitinase abc in the adult rat brain. Exp Brain Res 121: 300–310. 9746136 10.1007/s002210050463

[35] Thuret S, Moon L, Gage F (2006) Therapeutic interventions after spinal cord injury. Nat Rev Neurosci 7: 628–643. 16858391 10.1038/nrn1955

[36] Kim Y, Lawler S, Nowicki M, Chiocca E, Friedman A (2009) A mathematical model of brain tumor: pattern formation of glioma cells outside the tumor spheroid core. J Theo Biol 260: 359–371. 10.1016/j.jtbi.2009.06.025 19596356

[37] Swanson K, Alvord E, Murray J (2003) Virtual resection of gliomas: Effect of extent of resection on recurrence. Math Comp Modelling 37: 1177–1190.

[38] Harpold H, Alvord EC Jr, Swanson K (2007) The evolution of mathematical modeling of glioma proliferation and invasion. J Neuropathol Exp Neurol 66: 1–9. 17204931 10.1097/nen.0b013e31802d9000

[39] Corwin D, Holdsworth C, Rockne R, Trister A, Mrugala M, et al. (2013) Toward patient-specific, biologically optimized radiation therapy plans for the treatment of glioblastoma. PLoS One 8.. 10.1371/journal.pone.0079115 PMC3827144 24265748

[40] Badoual M, Deroulers C, Aubert M, Grammaticos B (2010) Modelling intercellular communication and its effects on tumour invasion. Phys Biol 7: 046013. 21178241 10.1088/1478-3975/7/4/046013

[41] Gerin C, Pallud J, Grammaticos B, Mandonnet E, Deroulers C, et al (2012) Improving the time-machine: estimating date of birth of grade ii gliomas. Cell proliferation 45: 76–90. 22168136 10.1111/j.1365-2184.2011.00790.x PMC6496223

[42] Aubert M, Badoual M, Freol S, Christov C, Grammaticos B (2006) A cellular automaton model for the migration of glioma cells. Phys Biol 3: 93–100. 16829695 10.1088/1478-3975/3/2/001

[43] Hatzikirou H, Basanta D, Simon M, Schaller K, Deutsch A (2010) 'go or grow': the key to the emergence of invasion in tumour progression? Math Med Biol 27: 255–281. 20610469 10.1093/imammb/dqq011

[44] Chauviere A, Preziosi L, Byrne H (2010) A model of cell migration within the extracellular matrix based on a phenotypic switching mechanism. Math Med Biol 27: 255–281. 19942606 10.1093/imammb/dqp021

[45] Pham K, Chauviere A, Hatzikirou H, Li X, Byrne H, et al.. (2011) Density-dependent quiescence in glioma invasion: instability in a simple reaction-diffusion model for the migration/proliferation dichotomy. Journal of Biological dynamics: doi: 10.1080/17513758.2011.590610. 10.1080/17513758.2011.590610 PMC3623708 22873675

[46] Kim Y (2013) Regulation of cell proliferation and migration in glioblastoma: New therapeutic approach. Frontiers in Molecular and Cellular Oncology 3: 53. 10.3389/fonc.2013.00053 PMC3600576 23508546

[47] Kim Y, Roh S (2013) A hybrid model for cell proliferation and migration in glioblastoma. Discrete and Continuous Dynamical Systems-B 18: 969–1015.

[48] Lowengrub J, Frieboes H, Jin F, Chuang Y, Li X, et al (2010) Nonlinear modelling of cancer: bridging the gap between cells and tumours. Nonlinearity 23: R1. 20808719 10.1088/0951-7715/23/1/r01 PMC2929802

[49] Rejniak K, Anderson A (2011) Hybrid models of tumor growth. WIRES Syst Biol Med 3: 115–125. 10.1002/wsbm.102 PMC3057876 21064037

[50] Friedman A, Tian J, Fulci G, Chiocca E, Wang J (2006) Glioma virotherapy: effects of innate immune suppression and increased viral replication capacity. Cancer Res 66: 2314–9. 16489036 10.1158/0008-5472.CAN-05-2661

[51] Muir E, Fyfe I, Gardiner S, Li L, Warren P, et al (2010) Modification of N-glycosylation sites allows secretion of bacterial chondroitinase ABC from mammalian cells. J Biotechnol 145: 103–10. 19900493 10.1016/j.jbiotec.2009.11.002 PMC2809921

[52] Gladson C (1999) The extracellular matrix of gliomas: modulation of cell function. J Neuropathol Exp Neurol 58: 1029–1040. 10515226 10.1097/00005072-199910000-00001

[53] Bellail A, Hunter S, Brat D, Tan C, Meir EV (2004) Microregional extracellular matrix heterogeneity in brain modulates glioma cell invasion. Int J Biochem Cell Biol 36: 1046–1069. 15094120 10.1016/j.biocel.2004.01.013

[54] Kim Y, Friedman A (2010) Interaction of tumor with its microenvironment: A mathematical model. Bull Math Biol 72: 1029–1068. 19908100 10.1007/s11538-009-9481-z

[55] Kim Y, Stolarska M, Othmer H (2011) The role of the microenvironment in tumor growth and invasion. Prog Biophys Mol Biol 106: 353–379. 21736894 10.1016/j.pbiomolbio.2011.06.006 PMC3156881

[56] Markert J, Liechty P, Wang W, Gaston S, Braz E, et al (2009) Phase ib trial of mutant herpes simplex virus G207 inoculated pre-and post-tumor resection for recurrent GBM. Mol Ther 17: 199–207. 18957964 10.1038/mt.2008.228 PMC2834981

[57] Davis F, McCarthy B (2001) Current epidemiological trends and surveillance issues in brain tumors. Expert Rev Anticancer Ther 1: 395–401. 12113106 10.1586/14737140.1.3.395

[58] Ozduman K, Wollmann G, Piepmeier J, van den Pol A (2008) Systemic vesicular stomatitis virus selectively destroys multifocal glioma and metastatic carcinoma in brain. J Neurosci 28: 1882–93. 18287505 10.1523/JNEUROSCI.4905-07.2008 PMC6671450

[59] Pardridge W (2002) Drug and gene delivery to the brain: the vascular route. Neuron 36: 555–8. 12441045 10.1016/s0896-6273(02)01054-1

[60] Pardridge W (2007) Blood-brain barrier delivery. Drug Discov Today 12: 54–61. 17198973 10.1016/j.drudis.2006.10.013

[61] Pope W, Sayre J, Perlina A, Villablanca J, Mischel P, et al (2005) Mr imaging correlates of survival in patients with high-grade gliomas. AJNR Am J Neuroradiol 26: 2466–74. 16286386 PMC7976216

[62] Parker J, Bauer D, Cody J, Markert J (2009) Oncolytic viral therapy of malignant glioma. Neurotherapeutics 6: 558–569. 19560745 10.1016/j.nurt.2009.04.011 PMC3980727

[63] Choucair A, Levin V, Gutin P, Davis R, Silver P, et al (1986) Development of multiple lesions during radiation therapy and chemotherapy in patients with gliomas. J Neurosurg 65: 654–8. 3021931 10.3171/jns.1986.65.5.0654

[64] Pasquier B, Pasquier D, N'Golet A, Panh M, Couderc P (1980) Extraneural metastases of astrocytomas and glioblastomas: clinicopathological study of two cases and review of literature. Cancer 45: 112–25. 6985826 10.1002/1097-0142(19800101)45:1<112::aid-cncr2820450121>3.0.co;2-9

[65] Ronnov-Jessen L, Petersen O, Bissell M (1996) Cellular changes involved in conversion of normal to malignant breast: importance of the stromal reaction. Physiol Rev 76: 69–125. 8592733 10.1152/physrev.1996.76.1.69

[66] Cheema T, Wakimoto H, Fecci P, Ning J, Kuroda T, et al.. (2013) Multifaceted oncolytic virus therapy for glioblastoma in an immunocompetent cancer stem cell model. Proc Nat Acad Sci USA ahead of print: doi:10.1073/pnas.1307935110 10.1073/pnas.1307935110 PMC3718117 23754388

[67] Whitley R, Markert J (2013) Viral therapy of glioblastoma multiforme. Proc Natl Acad Sci USA 110: 11672–11673. 23836634 10.1073/pnas.1310253110 PMC3718094

[68] Giese A, Bjerkvig R, Berens M, Westphal M (2003) Cost of migration: invasion of malignant gliomas and implications for treatment. J Clin Oncol 21: 1624–36. 12697889 10.1200/JCO.2003.05.063

[69] Beadle C, Assanah M, Monzo P, Vallee R, Rosenfield S, et al (2008) The role of myosin II in glioma invasion of the brain. Mol Biol Cell 19: 3357–3368. 18495866 10.1091/mbc.E08-03-0319 PMC2488307

[70] Lun X, Senger D, Alain T, Oprea A, Parato K, et al (2006) Effects of intravenously administered recombinant vesicular stomatitis virus (vsv(deltam51)) on multifocal and invasive gliomas. J Natl Cancer Inst 98: 1546–1557. 17077357 10.1093/jnci/djj413

[71] Pardridge W (2006) Molecular trojan horses for blood?brain barrier drug delivery. Current Opinion in Pharmacology 6: : 494–500. 10.1016/j.coph.2006.06.001 16839816

[72] Khuri F, Nemunaitis J, Ganly I, Arseneau J, Tannock I, et al (2000) A controlled trial of intratumoral ONYX-015, a selectively-replicating adenovirus, in combination with cisplatin and 5-fluorouracil in patients with recurrent head and neck cancer. Nat Med 6: 879–85. 10932224 10.1038/78638

[73] Pulkkanen K, Yla-Herttuala S (2005) Gene therapy for malignant glioma: current clinical status. Mol Ther 12: 585–98. 16095972 10.1016/j.ymthe.2005.07.357

[74] Puumalainen A, Vapalahti M, Agrawal R, Kossila M, Laukkanen J, et al (1998) Beta-galactosidase gene transfer to human malignant glioma in vivo using replication-deficient retroviruses and adenoviruses. Hum Gene Ther 9: 1769–1774. 9721087 10.1089/hum.1998.9.12-1769

[75] Harsh G, Deisboeck T, Louis D, Hilton J, Colvin MM, et al (2000) Thymidine kinase activation of ganciclovir in recurrent malignant gliomas: a gene-marking and neuropathological study. J Neurosurg 92: 804–11. 10794295 10.3171/jns.2000.92.5.0804

[76] Ram Z, Culver K, Oshiro E, Viola J, DeVroom H, et al. (1997) Therapy of malignant brain tumors by intratumoral implantation of retroviral vector-producing cells. Nat Med 3.. 10.1038/nm1297-1354 9396605

[77] Balachandran S, Porosnicu M, Barber G (2001) Oncolytic activity of vesicular stomatitis virus is effective against tumors exhibiting aberrant p53, Ras, or Myc function and involves the induction of apoptosis. J Virol 75: 3474–9. 11238874 10.1128/JVI.75.7.3474-3479.2001 PMC114141

[78] Connor J, Naczki C, Koumenis C, Lyles D (2004) Replication and cytopathic effect of oncolytic vesicular stomatitis virus in hypoxic tumor cells in vitro and in vivo. J Virol 78: 8960–70. 15308693 10.1128/JVI.78.17.8960-8970.2004 PMC506958

[79] Wollmann G, Tattersall P, van den Pol A (2005) Targeting human glioblastoma cells: comparison of nine viruses with oncolytic potential. J Virol 79: 6005–22. 15857987 10.1128/JVI.79.10.6005-6022.2005 PMC1091699

[80] Kaur B, Chiocca E, Cripe T (2012) Oncolytic hsv-1 virotherapy: clinical experience and opportunities for progress. Curr Pharm Biotechnol 13: 1842–1851. 21740359 10.2174/138920112800958814 PMC4233127

[81] Gotoh N (2008) Regulation of growth factor signaling by FRS2 family docking/scaffold adaptor proteins. Cancer Sci 99: 1319–25. 18452557 10.1111/j.1349-7006.2008.00840.x PMC11159094

[82] Black P, Agarwal P, Dinney C (2007) Targeted therapies in bladder cancer–an update. Urol Oncol 25: 433–8. 17826665 10.1016/j.urolonc.2007.05.011

[83] Green N, Morrison J, Hale S, Briggs S, Stevenson M, et al (2008) Retargeting polymer-coated adenovirus to the FGF receptor allows productive infection and mediates efficacy in a peritoneal model of human ovarian cancer. J Gene Med 10: 280–9. 18214996 10.1002/jgm.1121

[84] Kim J, Kim P, Kim S, Yun C (2012) Enhancing the therapeutic efficacy of adenovirus in combination with biomaterials. Biomaterials 33: 1838–50. 22142769 10.1016/j.biomaterials.2011.11.020 PMC3242832

[85] Verheije M, Lamfers M, Wurdinger T, Grinwis G, Gerritsen W, et al (2009) Coronavirus genetically redirected to the epidermal growth factor receptor exhibits effective antitumor activity against a malignant glioblastoma. J Virol 83: 7507–16. 19439466 10.1128/JVI.00495-09 PMC2708613

[86] Morrison J, Briggs S, Green N, Fisher K, Subr V, et al (2008) Virotherapy of ovarian cancer with polymer-cloaked adenovirus retargeted to the epidermal growth factor receptor. Mol Ther 16: 244–51. 18071336 10.1038/sj.mt.6300363

[87] Glasgow J, Everts M, Curiel D (2006) Transductional targeting of adenovirus vectors for gene therapy. Cancer Gene Ther 13: 830–44. 16439993 10.1038/sj.cgt.7700928 PMC1781516

[88] Nicklin S, Wu E, Nemerow G, Baker A (2005) The influence of adenovirus fiber structure and function on vector development for gene therapy. Mol Ther 12: 384–93. 15993650 10.1016/j.ymthe.2005.05.008

[89] Mok W, Stylianopoulos T, Boucher Y, Jain R (2009) Mathematical modeling of herpes simplex virus distribution in solid tumors: implications for cancer gene therapy. Clin Cancer Res 15: 2352–60. 19318482 10.1158/1078-0432.CCR-08-2082 PMC2872130

[90] Kim Y, Stolarska M, Othmer H (2007) A hybrid model for tumor spheroid growth in vitro I: Theoretical development and early results. Math Models Methods in Appl Scis 17: 1773–1798.

[91] Stolarska M, Kim Y, Othmer H (2009) Multiscale models of cell and tissue dynamics. Phil Trans Roy Soc A 367: 3525–3553. 19657010 10.1098/rsta.2009.0095 PMC3263796

[92] ODonoghue J, Bardies M, Wheldon T (1995) Relationships between tumor size and curability for uniformly targeted therapy with beta-emitting radionuclides. J Nucl Med 36: 1902–9. 7562062

[93] Gu W, Fu S, Wang Y, Li Y, Lu H, et al (2009) Chondroitin sulfate proteoglycans regulate the growth, differentiation and migration of multipotent neural precursor cells through the integrin signaling pathway. BMC Neurosci 10: 1–15. 19845964 10.1186/1471-2202-10-128 PMC2773784

[94] Silver DJ, Siebzehnrubl FA, Schildts MJ, Yachnis AT, Smith GM, et al (2013) Chondroitin sulfate proteoglycans potently inhibit invasion and serve as a central organizer of the brain tumor microenvironment. The Journal of Neuroscience 33: 15603–15617. 24068827 10.1523/JNEUROSCI.3004-12.2013 PMC3782629

[95] Fryer H, Kelly G, Molinaro L, Hockfield S (1992) The high molecular weight cat-301 chondroitin sulfate proteoglycan from brain is related to the large aggregating proteoglycan from cartilage, aggrecan. Journal of Biological Chemistry 267: 9874–9883. 1374409

[96] Heinegard D, Paulsson M, Inerot S, Carlstrom C (1981) A novel low-molecular weight chondroitin sulphate proteoglycan isolated from cartilage. Biochem J 197: 355–366. 6798963 10.1042/bj1970355 PMC1163134

[97] Dutt S, Kléber M, Matasci M, Sommer L, Zimmermann DR (2006) Versican v0 and v1 guide migratory neural crest cells. Journal of biological chemistry 281: 12123–12131. 16510447 10.1074/jbc.M510834200

[98] Dutt S, Matasci M, Sommer L, Zimmermann DR (2006) Guidance of neural crest cell migration: the inhibitory function of the chondroitin sulfate proteoglycan, versican. Scientific World Journal 6: 1114–7. 16964367 10.1100/tsw.2006.219 PMC5917207

[99] Viapiano MS, Bi WL, Piepmeier J, Hockfield S, Matthews RT (2005) Novel tumor-specific isoforms of behab/brevican identified in human malignant gliomas. Cancer research 65: 6726–6733. 16061654 10.1158/0008-5472.CAN-05-0585

[100] Bignami A, Hosley M, Dahl D (1993) Hyaluronic acid and hyaluronic acid-binding proteins in brain extracellular matrix. Anat Embryol 188: 419–33. 7508695 10.1007/BF00190136

[101] Dahl D, Chi N, Miles L, Nguyen B, Bignami A (1982) Glial fibrillary acidic (GFA) protein in schwann cells: fact or artefact? J Histochem Cytochem 30: 912–918. 6182187 10.1177/30.9.6182187

