# Supplementary material for: Choindroitinase ABC I-Mediated Enhancement of Oncolytic Virus Spread and Anti Tumor Efficacy: A Mathematical Model
Source: PLoS One. 2014 Jul 21;9(7):e102499. doi: 10.1371/journal.pone.0102499 (PMC4105445; doi:10.1371/journal.pone.0102499)
Supplement: Text S1 — Detailed description of the models and parameters used. (PDF) [file pone.0102499.s001.pdf]

# Supporting Material

## Choindroitinase ABC I-mediated enhancement of oncolytic virus spread and anti tumor efficacy: A mathematical model

Yangjin Kim, Hyun Geun Lee, Nina Dmitrieva, Junseok Kim, Balveen Kaur, and Avner Friedman

# S1. Detailed description of the models and parameters used

## Parameter Estimation

*Random motility of virus ($D_v, D_{PBS}$), controlling parameter ($\lambda_{51}$), Inhibition parameter of virus motility from ECM ($K_E$)*: Mok *et al.* [1] estimated the effective diffusion coefficient of HSV (herpes simplex virus) particles in tumors with high collagen content, $5 \times 10^{-10}$ $cm^2/s$ (=$1.8 \times 10^{-4}$ $mm^2/h$). Darcy permeability ($K$) of HSTS26T tumors led to the calculated diffusion coefficient of $2 \times 10^{-9}$ $cm^2/s$ and $2 \times 10^{-10}$ $cm^2/s$ for $K = 50$ $nm^2$ [2] and $K = 3$ $nm^2$ [3], respectively. Friedman et al [4] used high values of diffusion coefficient, $3.6 \times 10^{-2}$ $mm^2/h$. We take $D_{PBS} = 1.08 \times 10^{-7}$ $cm^2/s$ (=$3.89 \times 10^{-2}$ $mm^2/h$). We note here that the effective random motility of virus $D_v$ depends ECM concentrations, especially at the invading front where virus face both high and low concentrations of ECM in the the absence and presence of Chase-ABC. The random motility of virus and viral penatration differ significantly on the periphery and center of the tumor spheroids as illustrated in [5] and ECM concentrations at the invading front fluctuates due to virus infiltration and Chase-ABC molecules, leading to varying $D_v$. Based on experimental observations in [5], we take $\lambda_{51} = 2.25 \times 10^{-10}$ $mm^3/g$ and $K_E = 1.0 \times 10^{-8}$ $mm^3/g$. By assuming fluctuating averaged CSPG ECM concentration $E^s_{ave} = (0 - 2.6)$ $mg/ml$ [6] and with parameters $\lambda_{51}, K_E$ taken above, we get the dimensionless scaling factor $\frac{\lambda_{51}}{K_E + E^s_{ave}} = 8.6 \times 10^{-5} - 2.25 \times 10^{-2}$, leading to the effective diffusion coefficient $D_v = \frac{\lambda_{51}}{K_E + E^s_{ave}} D_{PBS}$ $=9.29 \times 10^{-12} - 2.43 \times 10^{-9}$ $cm^2/s$, which is rather consistent with experimental data in Mok *et al.* [1].

*Diffusion coefficient of Chase-ABC ($D_C$)*: Diffusion coefficient of typical ECM-degrading molecules such as MMPs is small. For instance, Saffarian *et al.* [7] estimated the diffusion coefficient of MMP-1 to be $(6.7 \pm 1.5) \times 10^{-9}$ $cm^2/s$ for inactive mutant MMP-1 and $(8 \pm 1.5) \times 10^{-9}$ $cm^2/s$ for wide-type (activated) MMP-1. In our simulation, we take $D_C = 1.08 \times 10^{-9}$ $cm^2/s$ (=$3.89 \times 10^{-4}$ $mm^2/h$).

*Proliferation rate of tumor cells ($\lambda$)*: Friedman *et al.* [4] used $\lambda = 2.0 \times 10^{-2}$ $h^{-1}$. We take $\lambda = 0.1536 = 1.54 \times 10^{-1}$ $h^{-1}$.

*Infected cell lysis rate ($\delta$)*: Friedman *et al.* [4] used $\delta = \frac{1}{18\ h} = 0.0556$ $h^{-1}$. We take $\delta = 0.012 = 1.2 \times 10^{-2}$ $h^{-1}$.

*Infection rate ($\beta$)*: We take $\beta = 7.0 \times 10^{-10}$ $(mm^3/h\ virus)$ from [4].

*Removal rate of dead cells ($\mu$)*: Friedman *et al.* [4] used $\mu = \frac{1}{48\ h} = 0.0208$ $h^{-1}$. We take $\mu = 0.012 = 1.2 \times 10^{-2}$ $h^{-1}$.

*Clearance rate of viruses ($\gamma$)*: Friedman *et al.* [4] used $\gamma = 2.5 \times 10^{-2}$ $h^{-1}$. Mok *et al.* [8] used $\gamma = 4.8 \times 10^{-5}$ $s^{-1} = 1.728 \times 10^{-1}$ $h^{-1}$ for free (interstitial) and bound virus. In the *in vitro* experiments in [1], the half-lives of HSV particles at 37$^o$C were ∼4 hours ($\sim 1.733 \times 10^{-1}$ $h^{-1}$) and ∼24 hours ($\sim 2.89 \times 10^{-2}$ $h^{-1}$) in tumor cell conditioned media and in PBS, respectively. However, as Mok *et al.* [1] noted, virus is internalized rapidly without much time for degradation. We found that the computational results is not sensitive to this parameters in several magnitudes as also noted in Wu *et*

*al.* [9]. We take rather small value $\gamma = 1.8 \times 10^{-3}\ h^{-1}$.

*Burst size of infected cells (b)*: We take $b = 50\ virus/cell$ from Friedman *et al.* [4].

*ECM production rate from uninfected tumor cells ($\lambda_{42}$)*: Kim *et al.* [10] took ECM remodeling/production rate of $5.0 \times 10^{-6}\ s^{-1} = 0.018\ h^{-1}$. We take $\lambda_{42} = 6.0 \times 10^{-3}\ h^{-1}$. Assuming uninfected cell density $x = (10^4 - 10^6)\ cells/mm^3$ [4,11] and $K_x = x^*, m_2 = 1$ in Table 1, and $\lambda_{42}$ above, we get the effective production rate by uninfected tumor cells $\lambda_{42} \frac{x^{m_2}}{K_x^{m_2} + x^{m_2}} = (5.45 \times 10^{-4} - 3 \times 10^{-3})\ h^{-1}$.

*ECM degradation rate ($\lambda_{41}$)*: Kim *et al.* [10] took $3.0 \times 10^8\ cm^3/(g.s)$ for ECM degradation rate from MMP secretion of tumor cells. We take $\lambda_{41} = 9.0 \times 10^1\ h^{-1}$. By assuming $C = (0.5 - 250)\ mU/ml$ [12–14] and with $K_C = C^*, m_1 = 1$ in Table 1, this estimated value $\lambda_{21}$ leads to the effective degradation rate $\lambda_{41} \frac{C^{m_1}}{K_C^{m_1} + C^{m_1}} = (30 - 89.6)\ h^{-1}$.

*Natural decay rate of Chase ($\lambda_{62}$)*: Kim *et al.* [10] took the natural decay rate of MMPs to be $5.0 \times 10^{-6}\ s^{-1} = 1.8 \times 10^{-2}\ h^{-1}$. The half-life of Chase-ABC (at $37^o C$) can vary significantly [15]. The half-life was 6 days in brain of injected rat [13] while it can be 2-3 weeks with trehalose or albumin (stabilizers) [16,17]. In the simulation, assuming the half-life of 115 hours, we take $\lambda_{62} = log(2)/115h = 6.0 \times 10^{-3}\ h^{-1}$.

*Secretion rate of ECM degrading enzyme ($\lambda_C$)*: This parameter value is largely unknown. We take $\lambda_C = 3.0 \times 10^1\ mU/(h.g)$, but also ran several trials (Figure 5 in the main text) in order to gauge the effect of this parameter.

## Nondimensionalization

Table 2 in the main text lists the reference values in the model. We define $L = 3\ mm$ following experimental setup in [5] and take the characteristic diffusion coefficient $D = 1.5 \times 10^{-5} cm^2/s$ so that $T = 1.67\ h$. We determine the reference values for $x, y, n, v, E, C$ as follows:

*Cell density ($x, y, n$):* Chicoine *et al.* took $5 \times 10^5\ cells/cm^3$ [18]. Mok *et al.* [1] used high value $7.23 \times 10^8\ cells/ml$ from Thurber and Wittrup [19]. Friedman *et al.* used $10^6\ cells/mm^3 = 10^9\ cells/cm^3$ from [11]. Following [4,11], we take $x^* = y^* = n^* = 10^6\ cells/mm^3$.

*Virus concentration ($v$):* We take $v^* = 2.2 \times 10^{11}\ virus/cm^3 = 2.2 \times 10^8\ virus/mm^3$ [4,11]. We also note that virus concentration can be calculated indirectly from plaque-forming unit (pfu). For instance, Mok *et al.* [1] estimated the total viral concentration from $1 \times 10^6$ pfu by using a factor of 50. In [5], $3 \times 10^5$ pfu of rHsvQ or OV-Chase were used.

*ECM density (CSPG ($E^*$), Tumor ECM ($\rho^*$)):* Various CSPG concentrations (0-500 $\mu g/ml$) and 5 $\mu g/ml$ laminin were used for the invasive and noninvasive coculture spot assays in a study of the role of CSPG in regulation of glioma invasion and CSPG was shown to be a potent activator of microglia in vivo and to play as a key organizer of the brain tumor microenvironment [20]. High molecular weight Cat-301 CNS CSPG from brain in the density of 1.4 $g/ml$ [21] was shown to have similar properties as aggrecan, the high molecular weight CSPG from cartilage with typical low-buoyant-densities 1.35-1.4$g/ml$ [22]. Using isoforms of one of major CSPG components, version (Intact versicans V1, V2, and amixture of V0 and V1) isolated from calf aorta, bovine spinal cord, and the spent culture medium of the human glioma cell line U251MG, respectively, Dutt *et al.* investigated the role of versican V0 and V1 in the range of 0-100 $\mu g/ml$ in regulation of neural crest cell migration. They found that even low levels of version V0/V1 ($> 25\ \mu g/ml$) can inhibit neural crest stem cell migration [23,24]. Also, total expression of noncleaved isoforms of BEHAB/Brevican was found to be >4-fold higher in human malignant gliomas compared with normal brain tissue [25]. Isolated versican from brain tissue was estimated to be $3mg/100g$ wet

tissue [26]. We take $E^* = 1.0\ mg/cm^3$. Glial HA-binding protein (GHAP), a brain-specific protein mainly localized in white matter, is present in high concentrations in CNS tissues, 8.2 $mg/100g$ in human white matter (wet tissue) [26] compared to the concentration of glial fibrillary acidic protein (GFAP) in buffered extracts of human spinal cord, 3.5 $mg/100g$ [27]. We take $\rho^*$=1.0 $mg/cm^3$.

*Concentration of Chase-ABC ($C^*$):* High-dose infusions of Chase $(2-1,000\ U/mL)$ is necessary in order to get diffusion of Chase-ABC into deep regions of the spinal cord when it is delivered intrathecally because of attendant dilution and overflow beyond the intrathecal space [17]. In a study of delivery of thermostabilized Chase for functional recovery after injury, Lee *et al.* [17] found that trahalose-assisted Chase in the concentration of 2 $U/0.5mL$ was enough to digest CSPG decorin. In a study of effects of Chase ABC on the morphology of neural precursor cells (NPCs) expanded in spheres, Gu *et al.* [14] used a wide concentration range of Chase ABC (0.5-50 $mU/mL$) in addition to 20 $ng/ml$ EGF and 20 $ng/ml$ bFGF. In a study of effect of Chase-ABC on acute and long-lasting changes in CSPG, injected Chase ABC at a concentration of 0.25 $U/\mu l$ led to significant degradation of ECM in the adult rat brain [12]. The protease-free Chase-ABC in the concentration of 50 $U/ml$ showed digestion of CSPGs around the injection site (leading to low GAG content (1,200-2,000 $\mu g/mg$) compared to high peak values ($4,000\ \mu g/mg$) with penicillinase-treatment) and promoted axon regeneration [13]. We take $C^* = 50\ mU/ml$.

We nondimensionalize the variables and parameters in the partial differential equations (A.1)-(A.9) as follows:

$$\begin{aligned}
&\bar{t} = \frac{t}{T},\ \bar{\mathbf{x}} = \frac{\mathbf{x}}{L},\ \bar{x} = \frac{x}{x^*},\ \bar{y} = \frac{y}{y^*},\ \bar{n} = \frac{n}{n^*},\ \bar{E} = \frac{E}{E^*},\ \bar{\rho} = \frac{\rho}{\rho^*},\ \bar{v} = \frac{v}{v^*},\ \bar{C} = \frac{C}{C^*},\\
&\bar{D}_{PBS} = D_{PBS}/D,\ \bar{D}_C = D_C/D,\ \bar{\lambda} = T\lambda,\ \bar{x}_0 = \frac{\bar{x}_0}{x^*},\ \bar{\beta} = Tv^*\beta,\ \bar{\beta}^\dagger = \frac{Tv^*\beta x^*}{y^*},\\
&\bar{\delta} = T\delta,\ \bar{\delta}^\dagger = \frac{T\delta y^*}{n^*},\ \bar{\mu} = T\mu,\ \bar{\lambda}_{41} = T\lambda_{41},\ \bar{K}_C = \frac{K_C}{C^*},\ \bar{\lambda}_{42} = T\lambda_{42},\ \bar{K}_x = \frac{K_x}{x^*},\\
&\bar{\rho}_0 = \frac{\bar{\rho}_0}{\rho^*},\ \bar{\lambda}_{51} = \frac{\lambda_{51}}{E^*},\ \bar{K}_E = \frac{K_E}{E^*},\ \bar{b} = b\frac{y^*}{v^*},\ \bar{\gamma} = T\gamma,\ \bar{\lambda}_{63} = TE^*\lambda_{63},\ \bar{\lambda}_{62} = T\lambda_{62},\\
&\bar{\lambda}_C = \frac{T\lambda_C y^*}{C^*}.
\end{aligned} \tag{1}$$

The governing equations in a dimensionless form are

$$\frac{\partial \bar{x}}{\partial \bar{t}} + \bar{\nabla}\cdot(\bar{x}\bar{\boldsymbol{u}}) = \bar{\lambda}\bar{x}(1-\bar{x}/\bar{x}_0) - \bar{\beta}\bar{x}\bar{v} \quad \text{in } \Omega(t) \tag{2}$$

$$\frac{\partial \bar{y}}{\partial \bar{t}} + \bar{\nabla}\cdot(\bar{y}\bar{\boldsymbol{u}}) = \bar{\beta}^{\dagger}\bar{x}\bar{v} - \bar{\delta}\bar{y}, \quad \text{in } \Omega(t) \tag{3}$$

$$\frac{\partial \bar{n}}{\partial \bar{t}} + \bar{\nabla}\cdot(\bar{n}\bar{\boldsymbol{u}}) = \bar{\delta}^{\dagger}\bar{y} - \bar{\mu}\bar{n} \quad \text{in } \bar{\Omega}(t), \tag{4}$$

$$\frac{\partial \bar{E}}{\partial \bar{t}} + \nabla\cdot(\bar{E}\bar{\boldsymbol{u}}) = -\frac{\bar{\lambda}_{41}\bar{E}\;\bar{C}^{m_1}}{K_C^{m_1}+\bar{C}^{m_1}} \quad \text{in } \bar{\Omega}(t), \tag{5}$$

$$\frac{\partial \bar{\rho}}{\partial \bar{t}} + \nabla\cdot(\bar{\rho}\bar{\boldsymbol{u}}) = \bar{\lambda}_{42}\frac{\bar{x}^{m_2}}{\bar{K}_x^{m_2}+\bar{x}^{m_2}}\bar{\rho}\left(1-\frac{\bar{\rho}}{\bar{\rho}_0}\right) \quad \text{in } \bar{\Omega}(t), \tag{6}$$

$$\frac{\partial \bar{v}}{\partial \bar{t}} = \bar{\nabla}\cdot\left(\frac{\bar{\lambda}_{51}}{\bar{K}_E+\bar{E}^s_{ave}}\bar{D}_{PBS}\bar{\nabla}\bar{v}\right) + \bar{b}\bar{\delta}\bar{y}I_{\Omega(t)} - \bar{\gamma}\bar{v}, \quad \text{in } \bar{\Omega}_0, \tag{7}$$

$$\frac{\partial \bar{C}}{\partial \bar{t}} = \bar{\nabla}\cdot(\bar{D}_C\bar{\nabla}\bar{C}) + \bar{g}(\bar{v},\bar{y},\Omega) - \bar{\lambda}_{63}\bar{E}\bar{C} - \bar{\lambda}_{62}\bar{C}, \quad \text{in } \bar{\Omega}_0, \tag{8}$$

$$-\bar{\Delta}\bar{\boldsymbol{u}} + \bar{\nabla}\bar{p} = \bar{\eta}\bar{\nabla}\left(\bar{\lambda}(1-\bar{x}/\bar{x}_0) - \bar{\mu}\bar{n} + \bar{\lambda}_{42}\frac{\bar{x}^{m_2}}{\bar{K}_x^{m_2}+\bar{x}^{m_2}}\bar{\rho}\big(1-\frac{\bar{\rho}}{\bar{\rho}_0}\big) - \bar{\lambda}_{71}\frac{\bar{\lambda}_{41}\bar{E}\;\bar{C}^{m_1}}{\bar{K}_c^{m_1}+\bar{C}^{m_1}}\right), \tag{9}$$

$$div\ \bar{\boldsymbol{u}} = \bar{\lambda}\bar{x}(1-\bar{x}/\bar{x}_0) - \bar{\mu}\bar{n} + \bar{\lambda}_{42}\frac{\bar{x}^{m_2}}{\bar{K}_x^{m_2}+\bar{x}^{m_2}}\bar{\rho}\big(1-\frac{\bar{\rho}}{\bar{\rho}_0}\big) - \bar{\lambda}_{71}\frac{\bar{\lambda}_{41}\bar{E}\;\bar{C}^{m_1}}{\bar{K}_c^{m_1}+\bar{C}^{m_1}}, \tag{10}$$

$$\frac{d\bar{X}}{d\bar{t}} = \bar{\boldsymbol{u}}, \text{ on } \Gamma(t) \tag{11}$$

$$\frac{\partial \bar{v}}{\partial \bar{\nu}} = 0, \text{ on } \partial\Omega_0, \tag{12}$$

$$\frac{\partial \bar{C}}{\partial \bar{\nu}} = 0, \text{ on } \partial\Omega_0. \tag{13}$$

*Note that $\bar{\beta}^{\dagger} = \bar{\beta}$, $\bar{\delta}^{\dagger} = \bar{\delta}$ under the assumption of $x^* = y^* = n^*$; otherwise $\bar{\beta}^{\dagger}, \bar{\delta}^{\dagger}$ from $\bar{\beta}, \bar{\delta}$, respectively.

## Numerical scheme

Equations (2)-(13) were solved using multigrid method [28] and vanka relaxation method [29].

Velocities are defined at cell boundaries while $x$, $y$, $n$, $E$, $\rho$, $v$, $C$, and $p$ are defined at the cell centers. Let a computational domain be partitioned in Cartesian geometry into a uniform mesh with mesh spacing $h$. The center of each cell, $\Omega_{ij}$, is located at $(x_i, y_j) = ((i-0.5)h, (j-0.5)h)$ for $i = 1, \cdots, N_x$ and $j = 1, \cdots, N_y$. $N_x$ and $N_y$ are the numbers of cells in $x$ and $y$-directions, respectively. The cell vertices are located at $(x_{i+\frac{1}{2}}, y_{j+\frac{1}{2}}) = (ih, jh)$.

Let $\Delta t$ be a time step and $k$ be a time step index. At the beginning of each time step, given $x^k$, $y^k$, $n^k$, $E^k$, $\rho^k$, $v^k$, $C^k$, and $\boldsymbol{u}^k$, we want to find $x^{k+1}$, $y^{k+1}$, $n^{k+1}$, $E^{k+1}$, $\rho^{k+1}$, $v^{k+1}$, $C^{k+1}$, $\boldsymbol{u}^{k+1}$, and $p^{k+1}$

which solve the following temporal discretization of equations (2)-(10):

$$\frac{x^{k+1}-x^k}{\Delta t}+\nabla_d\cdot(x^k\boldsymbol{u}^k)=\lambda x^k(1-x^k/x_0)-\beta x^k v^k,\quad \text{in } \Omega(t) \tag{14}$$

$$\frac{y^{k+1}-y^k}{\Delta t}+\nabla_d\cdot(y^k\boldsymbol{u}^k)=\beta x^k v^k-\delta y^k,\quad \text{in } \Omega(t) \tag{15}$$

$$\frac{n^{k+1}-n^k}{\Delta t}+\nabla_d\cdot(n^k\boldsymbol{u}^k)=\delta y^k-\mu n^k,\quad \text{in } \Omega(t) \tag{16}$$

$$\frac{E^{k+1}-E^k}{\Delta t}+\nabla_d\cdot(E^k\boldsymbol{u}^k)=-\frac{\lambda_{41}E^k\,(C^k)^{m_1}}{K_C^{m_1}+(C^k)^{m_1}},\quad \text{in } \Omega(t), \tag{17}$$

$$\frac{\rho^{k+1}-\rho^k}{\Delta t}+\nabla_d\cdot(\rho^k\boldsymbol{u}^k)=\lambda_{42}\frac{(x^{k+1})^{m_2}}{K_x^{m_2}+(x^{k+1})^{m_2}}\rho^k\left(1-\frac{\rho^k}{\rho_0}\right),\quad \text{in } \Omega(t) \tag{18}$$

$$\frac{v^{k+1}-v^k}{\Delta t}=\nabla_d\cdot\left(\frac{\lambda_{51}}{K_E+(E^{k+1})^s_{ave}}D_{PBS}\nabla_d v^{k+1}\right)+b\delta y^{k+1}I_{\Omega(t)}-\gamma v^{k+1},\quad \text{in } \Omega_0, \tag{19}$$

$$\frac{C^{k+1}-C^k}{\Delta t}=\nabla_d\cdot(D_C\nabla C^{k+1})+g(v^{k+1},y^{k+1},\Omega)-\lambda_{63}E^{k+1}C^{k+1}-\lambda_{62}C^{k+1},\quad \text{in } \Omega_0, \tag{20}$$

$$\begin{aligned}-\Delta_d\boldsymbol{u}^{k+1}+\nabla_d p^{k+1}=\eta\nabla_d\Bigg(&\lambda x^{k+1}(1-x^{k+1}/x_0)-\mu n^{k+1}\\ &+\lambda_{42}\frac{(x^{k+1})^{m_2}}{K_x^{m_2}+(x^{k+1})^{m_2}}\rho^{k+1}\big(1-\frac{\rho^{k+1}}{\rho_0}\big)-\lambda_{71}\frac{\lambda_{41}E^{k+1}\,(C^{k+1})^{m_1}}{K_c^{m_1}+(C^{k+1})^{m_1}}\Bigg),\end{aligned} \tag{21}$$

$$\begin{aligned}div\ \boldsymbol{u}^{k+1}=&\lambda x^{k+1}(1-x^{k+1}/x_0)-\mu n^{k+1}+\lambda_{42}\frac{(x^{k+1})^{m_2}}{K_x^{m_2}+(x^{k+1})^{m_2}}\rho^{k+1}\big(1-\frac{\rho^{k+1}}{\rho_0}\big)\\ &-\lambda_{71}\frac{\lambda_{41}E^{k+1}\,(C^{k+1})^{m_1}}{K_c^{m_1}+(C^{k+1})^{m_1}}.\end{aligned} \tag{22}$$

The outline of the main procedures in one time step is:

*Step 1.* Initialize $x^0$, $y^0$, $n^0$, $E^0$, $\rho^0$, $v^0$, $C^0$, and $\boldsymbol{u}^0$.

*Step 2.* Solve $x^{k+1}$, $y^{k+1}$, $n^{k+1}$, $E^{k+1}$, and $\rho^{k+1}$. The resulting finite difference equations (14)-(18) are written out explicitly. They take the form

$$\frac{x^{k+1}}{\Delta t}=\frac{x^k}{\Delta t}-\nabla\cdot(x^k\boldsymbol{u}^k)+\lambda x^k(1-x^k/x_0)-\beta x^k v^k,\quad \text{in } \Omega(t) \tag{23}$$

$$\frac{y^{k+1}}{\Delta t}=\frac{y^k}{\Delta t}-\nabla\cdot(y^k\boldsymbol{u}^k)+\beta x^n v^k-\delta y^n,\quad \text{in } \Omega(t) \tag{24}$$

$$\frac{n^{k+1}}{\Delta t}=\frac{n^k}{\Delta t}-\nabla\cdot(n^k\boldsymbol{u}^k)+\delta y^n-\mu n^n,\quad \text{in } \Omega(t), \tag{25}$$

$$\frac{E^{k+1}}{\Delta t}=\frac{E^k}{\Delta t}-\nabla_d\cdot(E^k\boldsymbol{u}^k)-\frac{\lambda_{41}E^k\,(C^k)^{m_1}}{K_C^{m_1}+(C^k)^{m_1}},\quad \text{in } \Omega(t) \tag{26}$$

$$\frac{\rho^{k+1}}{\Delta t}=\frac{\rho^k}{\Delta t}-\nabla_d\cdot(\rho^k\boldsymbol{u}^k)+\lambda_{42}\frac{(x^{k+1})^{m_2}}{K_x^{m_2}+(x^{k+1})^{m_2}}\rho^k\left(1-\frac{\rho^k}{\rho_0}\right),\quad \text{in } \Omega(t), \tag{27}$$

where the advection term, $\nabla \cdot (x^k \boldsymbol{u}^k)$, is defined by:

$$((xu)_x + (xw)_y)_{ij}^k = \frac{u_{i+\frac{1}{2},j}^k(x_{i+1,j}^k + x_{ij}^k) - u_{i-\frac{1}{2},j}^k(x_{ij}^k + x_{i-1,j}^k)}{2h} \tag{28}$$

$$+\frac{w_{i,j+\frac{1}{2}}^k(x_{i,j+1}^k + x_{ij}^k) - w_{i,j-\frac{1}{2}}^k(x_{ij}^k + x_{i,j-1}^k)}{2h}, \tag{29}$$

where $u$ and $w$ are horizontal and vertical velocity components, respectively. The quantities $\nabla \cdot (y^k \boldsymbol{u}^k)$, $\nabla \cdot (n^k \boldsymbol{u}^k)$, $\nabla \cdot (E^k \boldsymbol{u}^k)$, and $\nabla \cdot (\rho^k \boldsymbol{u}^k)$ are computed in a similar manner.

*Step 3.* The resulting implicit discrete system (19)-(20) is solved by a linear geometric multigrid method [28].

*Step 4.* The resulting implicit discrete system (21)-(22) is solved by a coupled block-implicit multigrid method [29].

A pointwise Gauss–Seidel relaxation scheme is used as the smoother in the linear geometric and coupled block-implicit multigrid methods.

These complete the one time step.

Program is written in *C*. Calculations were performed on a Intel Core i3 CPU (3.20 GHz) with 2 GB of RAM.

# References


1. Mok W, Stylianopoulos T, Boucher Y, Jain R (2009) Mathematical modeling of herpes simplex virus distribution in solid tumors: implications for cancer gene therapy. Clin Cancer Res 15: 2352-60.

2. Netti P, Berk D, Swartz M, Grodzinsky A, Jain R (2000) Role of extracellular matrix assembly in interstitial transport in solid tumors. Cancer Res 60: 2497-2503.

3. Griffon-Etienne G, Boucher Y, Brekken C, Suit H, Jain R (1999) Taxane-induced apoptosis decompresses blood vessels and lowers interstitial fluid pressure in solid tumors: clinical implications. Cancer Res 59: 3776–82.

4. Friedman A, Tian J, Fulci G, Chiocca E, Wang J (2006) Glioma virotherapy: effects of innate immune suppression and increased viral replication capacity. Cancer Res 66: 2314-9.

5. Dmitrieva N, Yu L, Viapiano M, Cripe T, Chiocca E, et al. (2011) Choindroitinase ABC I-mediated enhancement of oncolytic virus spread and antitumor efficacy. Clin Cancer Res 17: 1362–72.

6. Stein A, Demuth T, Mobley D, Berens M, Sander L (2007) A mathematical model of glioblastoma tumor spheroid invasion in a three-dimensional in vitro experiment. Biophys J 92: 356-65.

7. Saffarian S, Collier I, Marmer B, Elson E, Goldberg G (2004) Interstitial collagenase is a brownian ratchet driven by proteolysis of collagen. Science 306: 108-11.

8. Mok W, Boucher Y, Jain R (2007) Matrix metalloproteinases-1 and -8 improve the distribution and efficacy of an oncolytic virus. Cancer Res 67: 10664-10668.

9. Wu J, Byrne H, Kirn D, Wein L (2001) Modeling and analysis of a virus that replicates selectively in tumor cells. Bull Math Biol 63: 731-68.

10. Kim Y, Lawler S, Nowicki M, Chiocca E, Friedman A (2009) A mathematical model of brain tumor : pattern formation of glioma cells outside the tumor spheroid core. J Theo Biol 260: 359-371.

11. ODonoghue J, Bardies M, Wheldon T (1995) Relationships between tumor size and curability for uniformly targeted therapy with beta-emitting radionuclides. J Nucl Med 36: 1902–9.

12. Bruckner G, Bringmann A, Hartig W, Koppe G, Delpech B, et al. (1998) Acute and long-lasting changes in extracellular-matrix chondroitin-sulphate proteoglycans induced by injection of chondroitinase abc in the adult rat brain. Exp Brain Res 121: 300-310.

13. Lin R, Kwok J, Crespo D, Fawcett J (2008) Chondroitinase abc has a long-lasting effect on chondroitin sulphate glycosaminoglycan content in the injured rat brain. J Neurochem 104: 400-408.

14. Gu W, Fu S, Wang Y, Li Y, Lu H, et al. (2009) Chondroitin sulfate proteoglycans regulate the growth, differentiation and migration of multipotent neural precursor cells through the integrin signaling pathway. BMC Neurosci 10: 1–15.

15. Zhao R, Muir E, Alves J, Rickman H, Allan A, et al. (2011) Lentiviral vectors express chondroitinase ABC in cortical projections and promote sprouting of injured corticospinal axons. J Neurosci Methods 201: 228-38.

16. Chau C, Shum D, Li H, Pei J, Lui Y, et al. (2004) Chondroitinase ABC enhances axonal regrowth through Schwann cell-seeded guidance channels after spinal cord injury. FASEB J 18: 194–196.

17. Lee H, McKeon R, Bellamkonda R (2010) Sustained delivery of thermostabilized chabc enhances axonal sprouting and functional recovery after spinal cord injury. PNAS 107: 3340–3345.

18. Chicoine M, Madsen C, Silbergeld D (1995) Modification of human glioma locomotion in vitro by cytokines EGF, bFGF, PDGFbb, NGF, and TNF alpha. Neurosurgery 36: 1165-70; disbrain cancer.

19. Thurber G, Wittrup K (2008) Quantitative spatiotemporal analysis of antibody fragment diffusion and endocytic consumption in tumor spheroids. Cancer Res 68: 3334–41.

20. Silver DJ, Siebzehnrubl FA, Schildts MJ, Yachnis AT, Smith GM, et al. (2013) Chondroitin sulfate proteoglycans potently inhibit invasion and serve as a central organizer of the brain tumor microenvironment. The Journal of Neuroscience 33: 15603–15617.

21. Fryer H, Kelly G, Molinaro L, Hockfield S (1992) The high molecular weight cat-301 chondroitin sulfate proteoglycan from brain is related to the large aggregating proteoglycan from cartilage, aggrecan. Journal of Biological Chemistry 267: 9874–9883.

22. Heinegard D, Paulsson M, Inerot S, Carlstrom C (1981) A novel low-molecular weight chondroitin sulphate proteoglycan isolated from cartilage. Biochem J 197: 355–366.

23. Dutt S, Kléber M, Matasci M, Sommer L, Zimmermann DR (2006) Versican v0 and v1 guide migratory neural crest cells. Journal of biological chemistry 281: 12123–12131.

24. Dutt S, Matasci M, Sommer L, Zimmermann DR (2006) Guidance of neural crest cell migration: the inhibitory function of the chondroitin sulfate proteoglycan, versican. Scientific World Journal 6: 1114–7.

25. Viapiano MS, Bi WL, Piepmeier J, Hockfield S, Matthews RT (2005) Novel tumor-specific isoforms of behab/brevican identified in human malignant gliomas. Cancer research 65: 6726–6733.

26. Bignami A, Hosley M, Dahl D (1993) Hyaluronic acid and hyaluronic acid-binding proteins in brain extracellular matrix. Anat Embryol 188: 419–33.

27. Dahl D, Chi N, Miles L, Nguyen B, Bignami A (1982) Glial fibrillary acidic (gfa) protein in schwann cells: fact or artefact? J Histochem Cytochem 30: 912–918.

28. Trottenberg U, Oosterlee C, Schuller (2001) Multigrid. Academic Press.

29. Vanka S (1986) Block-implicit multigrid solution of Navier-Stokes equations in primitive variables. J Comp Phys 65: 138–158.
